# ITGB1 enhances the Radioresistance of human Non-small Cell Lung Cancer Cells by modulating the DNA damage response and YAP1-induced Epithelial-mesenchymal Transition

**DOI:** 10.7150/ijbs.52319

**Published:** 2021-01-18

**Authors:** Yuexian Li, Cheng Sun, Yonggang Tan, Heying Zhang, Yuchao Li, Huawei Zou

**Affiliations:** 1 Department of Oncology, Shengjing Hospital affiliated with China Medical University, Shenyang 110004, China.; 2 School and Hospital of Stomatology, China Medical University, Liaoning Provincial Key Laboratory of Oral Diseases.

**Keywords:** ITGB1, Yes-associated protein (YAP), radioresistance, epithelial-mesenchymal transition (EMT), non-small cell lung cancer (NSCLC)

## Abstract

**Objectives:** Radiotherapy has played a limited role in the treatment of non-small cell lung cancer (NSCLC) due to the risk of tumour radioresistance. We previously established the radioresistant non-small cell lung cancer (NSCLC) cell line H460R. In this study, we identified differentially expressed genes between these radioresistant H460R cells and their radiosensitive parent line. We further evaluated the role of a differentially expressed gene, ITGB1, in NSCLC cell radioresistance and as a potential target for improving radiosensitivity.

**Materials and Methods:** The radiosensitivity of NSCLC cells was evaluated by flow cytometry, colony formation assays, immunofluorescence, and Western blotting. Bioinformatics assay was used to identify the effect of ITGB1 and YAP1 expression in NSCLC tissues.

**Results:** ITGB1 mRNA and protein expression levels were higher in H460R than in the parental H460 cells. We observed lower clonogenic survival and cell viability and a higher rate of apoptosis of ITGB1-knockdown A549 and H460R cells than of wild type cells post-irradiation. Transfection with an ITGB1 short hairpin (sh) RNA enhanced radiation-induced DNA damage and G2/M phase arrest. Moreover, ITGB1 induced epithelial-mesenchymal transition (EMT) of NSCLC cells. Silencing ITGB1 suppressed the expression and intracellular translocation of Yes-associated protein 1 (YAP1), a downstream effector of ITGB1.

**Conclusions:** ITGB1 may induce radioresistance via affecting DNA repair and YAP1-induced EMT. Taken together, our data suggest that ITGB1 is an attractive therapeutic target to overcome NSCLC cell radioresistance.

## Introduction

Lung cancer is the leading cause of cancer death worldwide 1, 2. Approximately 85% of patients with lung cancer have non-small cell lung cancer (NSCLC); lung adenocarcinoma (LUAD), lung squamous cell carcinoma (LUSC), and large cell lung carcinoma are the most common NSCLC subtypes 3. Although radiotherapy is a core NSCLC treatment, tumour resistance and recurrence limit its success 4, 5. Understanding the mechanism underlying radioresistance may aid the development of more effective treatments.

We previously established the radioresistant NSCLC cell line H460R by dose-gradient irradiation to investigate the mechanism of NSCLC cell radioresistance 6. RNA-sequencing analysis was used to study the differentially expressed genes between H460R and its parental cells. Kyoto encyclopaedia of genes and genomes (KEGG) analysis showed that extracellular matrix (ECM)-receptor interaction, focal adhesion, and cell adhesion molecule signalling pathways were predominantly overexpressed. Increasing evidence indicates that interactions between the ECM and cancer cells play important roles in the development of resistance to ionizing radiation 7-9. Integrins, major cell-matrix adhesion receptors, comprise two transmembrane glycoproteins, α and β, which interact with ECM components to regulate cellular processes including proliferation, survival, cell death, metastasis, and therapy resistance 10-13. ITGB1 is the most common subchain in integrin heterodimers. Importantly, the integrin heterodimers that predominantly bind to the ECM proteins which are up-regulated in tumours contain the β1 subchain 14. Several studies have shown that ITGB1 is widely overexpressed in tumours such as the lung, breast, and colorectal tumours and plays an important role in their survival and metastatic potential 15-20. Other studies revealed that ITGB1 mediates tumour resistance to diverse anti-cancer drugs including erlotinib, bevacizumab, gemcitabine, and gefitinib 21-24. Meanwhile, ITGB1 inhibition enhances radiosensitivity and impairs DNA repair, thereby increasing residual DNA damage levels in head and neck squamous cell carcinoma (HNSCC) and pancreatic carcinoma cells 25, 26. However, little is known about its role in NSCLC cell radioresistance.

Yes-associated protein 1 (YAP1), a key downstream effector in the classical Hippo pathway, regulates organ development and tissue homeostasis 27. Overexpression of YAP1 occurs in several types of malignancies including breast, liver, colorectal, and lung cancer 28-31. Kras and YAP1 converge at the transcription factor FOS, activating a transcriptional program which regulates the epithelial-mesenchymal transition (EMT) and tumour survival 32. It has been revealed that YAP1 contributed to NSCLC migration and invasion by inducing the EMT program 33. EMT, originally observed during embryonic development, also plays an important role in tumour invasive behaviour, cancer stem cell properties, and resistance to chemotherapy, immunotherapy and radiotherapy in multiple cancers 34-36. Previously studies reported that NSCLC cells survived ionizing radiation treatment display the EMT phenotype. EMT-induced NSLCLC cells showed resistant to irradiation. More importantly, it has also been observed that radiotherapy may induce EMT *in vivo*, demonstrated by comparing surgically resected NSCLC specimens before and after radiotherapy 37-39. In addition, YAP1 enhances p73-dependant apoptosis in response to DNA damage in NSCLC 40, 41, and represents a pharmacological target to enhance the anti-tumour effects of DNA-damaging modalities for urothelial cell carcinoma treatment 42. Moreover, a recent study found that ITGB1-dependent cell adhesion was critical for supporting mesenchymal cell proliferation *in vivo* and vitro by controlling YAP1 signalling, rather than via the MAPK cascade 43. Physical attachment of cells to the ECM is essential for cell survival and growth 44. Cellular attachment to the ECM induces YAP1 nuclear localization through activation of Rho-GTPases or the FAK/Src/PI3K pathway 45, 46. Furthermore, ITGB1-dependent cell adhesion relies on YAP1 nuclear localization after dephosphorylation mediated by the large tumour suppressor gene 1 and 2 43. Herein, we explored if ITGB1 enhanced the radioresistance of human NSCLC cells through the regulation of YAP1.

DNA double-strand breaks (DNA-DSBs), one of the main types of ionizing radiation-induced damage, invoke various DNA repair mechanisms 47. Within minutes of the formation of radiation-induced DNA-DSBs, Ser139 of histone H2A family member X (H2AX) is rapidly phosphorylated around the DSB site; thereafter, the protein is known as phospho-histone H2AX (γH2AX), a DSB marker which is positively associated with radiosensitivity 48. Upon ionizing radiation-induced DNA damage, tumour cells primarily utilize two distinct kinase signalling cascades to repair DSBs: the ATM/CHK2 and ATR/CHK1 axes 49. The role of ITGB1 in radiation-induced DNA-DSBs and the signalling pathways through which ITGB1 exhibits its effects must be determined.

Thus, the purpose of our study was to investigate the potential role and mechanism of ITGB1 in the radioresistance of human lung cancer.

## Materials and Methods

### Transcriptomic and bioinformatic analysis

Transcriptome analysis was performed by Gene Denovo Biotechnology (Guangzhou, China). Briefly, total RNA was extracted from H460 or H460R cells using TRIzol™ reagent (Takara, Osaka, Japan). Triplicate RNA samples from independent groups were prepared for sequencing with a HiSeq 4000 (Illumina, San Diego, CA, USA) instrument. The primary bioinformatic analysis was carried out by Gene Denovo Biotechnology (Guangzhou, China). Human RNA sequencing data (1,102 cases, Workflow Type: HTSeq-Counts) and corresponding clinical information were downloaded from The Cancer Genome Atlas (TCGA) Genomic Data Commons data portal. RNA sequencing gene expression HTSeq-Count data and clinical data from 750 patients were used for further analysis. The associations between clinical pathologic features and ITGB1 expression were evaluated using the Wilcoxon signed-rank test and logistic regression. Clinicopathologic characteristics related to overall survival in patients with NSCLC were identified using the Cox regression and Kaplan-Meier methods. Multivariate Cox analysis was used to analyse the association of ITGB1 expression with survival, along with other clinical features (age, gender, stage, distant metastasis status, lymph node status, and histological subtype). The cut-off value for ITGB1 expression was determined based on its median value. Statistical analyses were performed using R software (V.3.6.3).

ITGB1 immunohistochemistry data were retrieved from the Human Protein Atlas database 50 to examine ITGB1 protein expression in NSCLC and healthy tissues. The GEPIA2 database 51 was used to conduct survival analyses based on gene expression levels and calculate hazard ratios (HRs) and 95% confidence intervals (CI); a log rank* P* <0.05 was considered the threshold for statistical significance. Additionally, the correlation between ITGB1 and YAP1 expression was analysed using Spearman's correlation coefficient in “correlation analysis”.

The genes that participate in DNA-DSB response pathways were downloaded from the PathCards database 52, and we constructed a protein-protein interaction (PPI) network for ITGB1 with these genes using the STRING database 53.

### Cell lines, cell culture, and irradiation protocol

The normal mammary epithelial cell line HBEC and four human NSCLC cell lines, A549, H460, H226, and H522, were purchased from the American Type Culture Collection (Manassas, VA, USA). LK2 cells were obtained from the Cell Bank of the Chinese Academy (Shanghai, China). H460R was previously established in our laboratory. All cells were cultured in RPMI-1640 medium (Sigma-Aldrich, Darmstadt, Germany) and supplemented with 10% foetal bovine serum (Clark Bioscience, Richmond, VA, USA) and 1% penicillin/streptomycin (Sigma-Aldrich, Darmstadt, Germany) at 37 °C in 5% CO_2_ in a humidified incubator. Cells were irradiated using a 6-MeV X-ray medical linear accelerator (Elekta Synergy, Elekta, Stockholm, Sweden) at a dose rate of 300 cGy/min (dose: 0 to 8 Gy) at room temperature.

### Lentiviral vector and small interfering RNA synthesis and transfection

The human ITGB1 shRNA (shITGB1) and empty vector lentiviral particles were constructed by GeneChem Company (Shanghai, China). The shITGB1 target sequence was 5′-CCTCCAGATGACATAGAAA-3′. Lentiviral particles expressing ITGB1 and the control lentiviral recombinants were obtained from Hanbio (Shanghai, China). Lentiviruses were transfected into A549, H522, and H460R cells, according to the manufacturer's protocol. The stably transfected cells were selected based on antibiotic resistance by culturing in 2 μg/ml puromycin (Solarbio, Beijing, China) for at least 2 weeks. The efficiency of transfection was assessed by quantitative real-time polymerase chain reaction (qRT-PCR) and western blot analyses. A small interfering RNA (siRNA) specifically targeting YAP1 and a control siRNA (scrambled) were designed and synthesized by GenePharma (Suzhou, China). Transfections were carried out using Lipofectamine 3000 reagent (Invitrogen, Carlsbad, CA, USA) according to the manufacturer's instructions. The transfected cells were harvested 72 h after transfection.

### RNA extraction, reverse transcription, and qRT-PCR

Total RNA was extracted from the cells using a TRIzol™ Plus Kit (Takara, Osaka, Japan) according to the manufacturer's instructions. Synthesized cDNA was prepared with a real-time PCR System (Life Technologies, Carlsbad, CA, USA) for qRT-PCR using an Applied Biosystems 7500 Real-Time PCR system with SYBR™ Green Master Mix (Takara, Osaka, Japan). The relative expression of *ITGB1* was determined with the 2^-△△Ct^ method after normalization to the expression of *GAPDH*. The primers for *ITGB1* were: forward, 5′-AAATGTAACCAACCGTAGC-3′ and reverse, 5′-GACAGGTCCATAAGGTAGTAGA-3′.

### Protein extraction and western blotting

Total proteins were extracted from cells using radioimmunoprecipitation assay lysis buffer (Beyotime, Shanghai, China). Total protein concentrations were quantified using a bicinchoninic acid assay kit (Beyotime, Shanghai, China). Nuclear and cytoplasmic proteins were extracted using the Minute™ Cytoplasmic and Nuclear Extraction Kit (Invent Biotechnologies, Beijing, China) according to the manufacturer's instructions. Equal amounts of proteins were boiled at 100 °C, separated by sodium dodecyl sulphate-polyacrylamide gel electrophoresis, and transferred onto polyvinylidene fluoride membranes (Millipore, Bedford, MA, USA). The membranes were blocked with 5% (w/v) skim milk at room temperature and immunoblotted at 4 °C overnight with primary antibodies against ITGB1 (Cat#12594-1-AP), E-cadherin (Cat#20874-1-AP), N-cadherin (Cat#22018-1-AP), CDC25c (Cat#16485-1-AP), GAPDH (Cat#60004-1-Ig), Histone H3 (Cat#17168-1-AP), YAP1(Cat#66900-1-Ig), and Zeb1 (Cat#21544-1-AP) from Proteintech (Chicago, IL, USA); γH2AX (Cat#ab81299), ATM(Cat#ab32420), CHK2 (Cat#ab109413), and phospho-CHK2 (Cat#ab32148) from Abcam, (San Diego, CA, USA); Snai (Cat#6032) phospho-CDC25c(Cat#AF3258) and phospho-ATM (Cat#AF4120) from Affinity Biosciences (Cincinnati, OH, USA); and vimentin (Cat#5741) from Cell Signaling Technology (Beverly, MA, USA). Then, the membranes were incubated with appropriate horseradish peroxidase (HRP)-conjugated secondary antibodies. GAPDH and Histone H3 were used as cytoplasmic and nuclear protein loading controls, respectively.

### Flow cytometric analysis

Cell apoptosis was measured using PE and FITC Annexin-V Apoptosis Detection Kits (BD Pharmingen, San Diego, CA, USA). The cells were irradiated with 0 or 8 Gy and cultured for an additional 48 h before incubation with PE- or FITC-labelled annexin V and 7-AAD or propidium iodide (PI) at room temperature in the dark for 15 min, according to the manufacturer's instructions.

Cell cycle analyses were performed using 50 µg/ml PI and 100 µg/ml DNase-free RNase A (Solarbio, Beijing, China). Twenty-four hours post-irradiation, cells were harvested with trypsin and washed with phosphate-buffered saline before fixation in 70% ice-cold ethanol at 4 °C for 12 h. After washing, the cell pellet was resuspended in PI staining buffer and incubated at 37°C for 30 min in the dark. Cell apoptosis and cell cycle status were analysed by flow cytometry (BD FACSCalibur, BD Biosciences, San Jose, CA, USA).

### Cell proliferation and colony-forming assay

After exposure to a single dose of radiation (8 Gy), cells were incubated for 24, 48, or 72 h. Then, we used Cell Counting Kit-8 (Beyotime, Shanghai, China) to determine cell viability post-irradiation. The absorbance was measured at a wavelength of 450 nm with a microplate reader (BioTek, Winooski, Vermont, USA). For the colony formation assay, 300 cells were seeded into 6-well plates and irradiated (0, 2, 4, 6, and 8 Gy) the next day. Two weeks later, the cells were fixed in 4% paraformaldehyde and stained with 0.1% crystal violet, and the number of colonies per dish was counted. The plating efficiency and surviving fraction were calculated as previously described 6.

### Immunofluorescence detection of γH2AX

Cells growing on glass coverslips were exposed to 0 or 8 Gy irradiation. The cells were then fixed in 4% paraformaldehyde 30 min, or 4, 8, or 24 h post-irradiation or without irradiation, and incubated with the primary phospho-γH2AX antibody (Ser139, Abcam, San Diego, CA, USA) and a secondary antibody conjugated to Cy3 (Beyotime, Shanghai, China) according to the manufacturer's protocol. We analysed the staining with a confocal laser scanning microscope (Nikon, Tokyo, Japan).

### Statistical analysis

Statistical analyses were performed with GraphPad Prism 7 software (San Diego, CA, USA). The statistical comparisons were made using Student's *t*-test, one-way analysis of variance (ANOVA), and two-way ANOVA. All data are expressed as the mean ± standard deviation (SD), and were repeated in at least three independent experiments. *P* values <0.05 (*) or <0.01 (**) were considered statistically significant.

## Results

### ITGB1 is overexpressed in H460R cells

**Figure 1:**
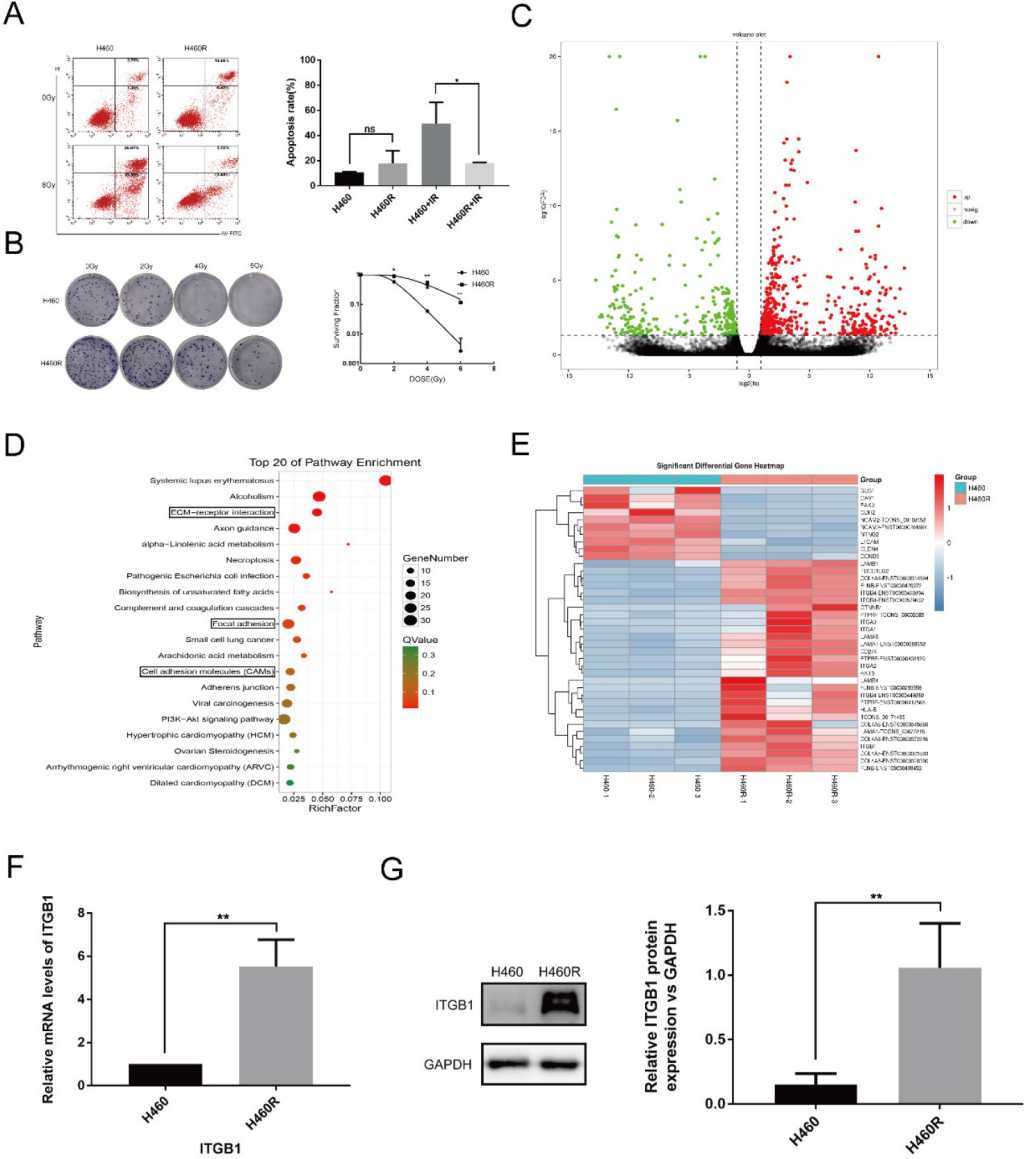
Amongst the differentially expressed genes in H460R and H460 cells, ITGB1 is overexpressed in H460R cells. A and B. Colony formation and cell apoptosis assays were used to determine the radiosensitivity of H460R and H460 cells. C. Volcano plot of differentially expressed genes in H460R and H460 cells. D. KEGG pathway analysis of the differentially expressed genes. E. Cluster analysis of the up-regulated (29) and down-regulated (10) genes in H460R cells compared with H460 cells involved in ECM-receptor interaction, focal adhesion and cell adhesion molecule signalling pathways. F and G. qRT-PCR and western blotting were performed to detected ITGB1 expression in H460R and H460 cells.

We used the clone formation assay and cell apoptosis analysis to confirm the radiosensitivity of H460R cells. Compared with the parental line, H460R had significantly higher colony formation ability and a lower rate of apoptosis post-irradiation (Fig. 1A-B). We next used RNA sequencing to investigate the differentially expressed genes in H460R and H460 cells. RNA-sequencing analysis was used to study the differentially expressed genes between H460R and its parental cells. As a result, we identified a total of 838 genes differed significantly at FDR < 0.05 with a fold change > 2, among which 508 genes were up-regulated and 330 genes were down-regulated in radio-resistant H460R cells compared with their radiosensitive parent cells. The complete list of the genes is shown in Supplementary Table S1. The differentially expressed genes are also represented in a volcano plot (Fig. 1C). KEGG analysis revealed that genes involved in ECM-receptor interaction, focal adhesion, and cell adhesion molecule signalling pathways were enriched amongst the differentially expressed genes (Fig. 1D). Unsupervised hierarchical clustering analysis was performed for these three signalling pathways (Fig. 1E).

We confirmed the differential expression of ITGB1 mRNA and protein in H460 and H460R cells by qRT-PCR and western blot analysis. We confirmed that ITGB1 was more highly expressed in H460R cells than in H460 cells (Fig. 1F and G).

### ITGB1 expression in H460R cells is associated with radiosensitivity

**Figure 2:**
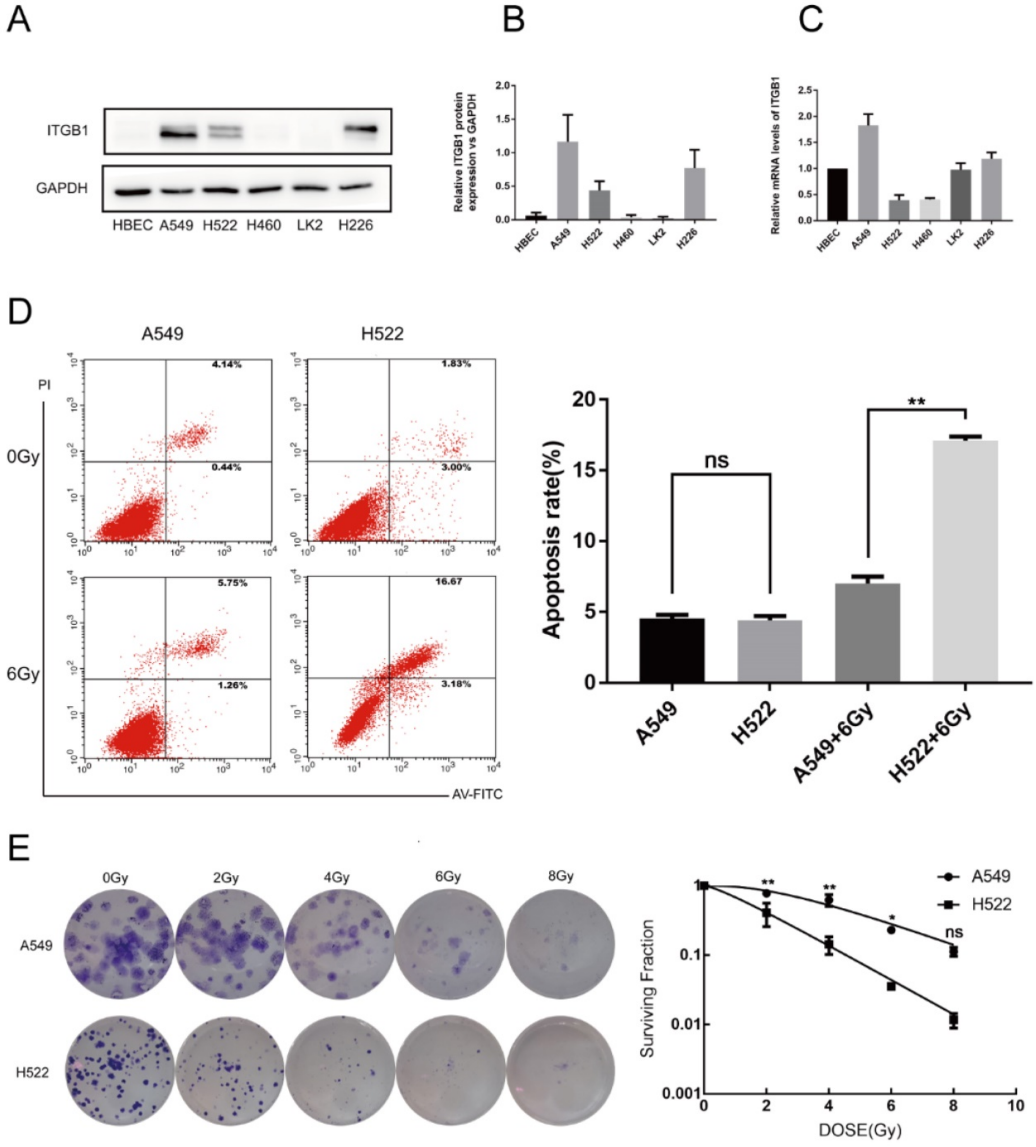
ITGB1 expression is associated with radiosensitivity. A-C. Western blotting and qRT-PCR were used to detect ITGB1 expression in the indicated cell lines. D. The apoptosis rates were determined by flow cytometry. E. Representative photographs of colony formation assays and the proportions of surviving A549 and H522 cells after irradiation with 0, 2, 4, 6, and 8 Gy.

To determine whether ITGB1 expression is associated with radiosensitivity in NSCLC, we measured ITGB1 expression in A549, H522, H460, LK2, and H226 cells (Fig. 2A-C). We selected the A549 and H522 cell lines for further study based on their high and low expression of ITGB1, respectively. Compared with H522 cells, A549 cells had a lower rate of apoptosis post-irradiation as assessed by flow cytometry (Fig. 2D). A549 cells also demonstrated significantly greater colony formation ability with better survival post-irradiation compared with H522 cells (Fig. 2E). These results suggest that ITGB1 overexpression is associated with radioresistance.

### ITGB1 expression negatively correlates with prognosis in patients with NSCLC

**Figure 3:**
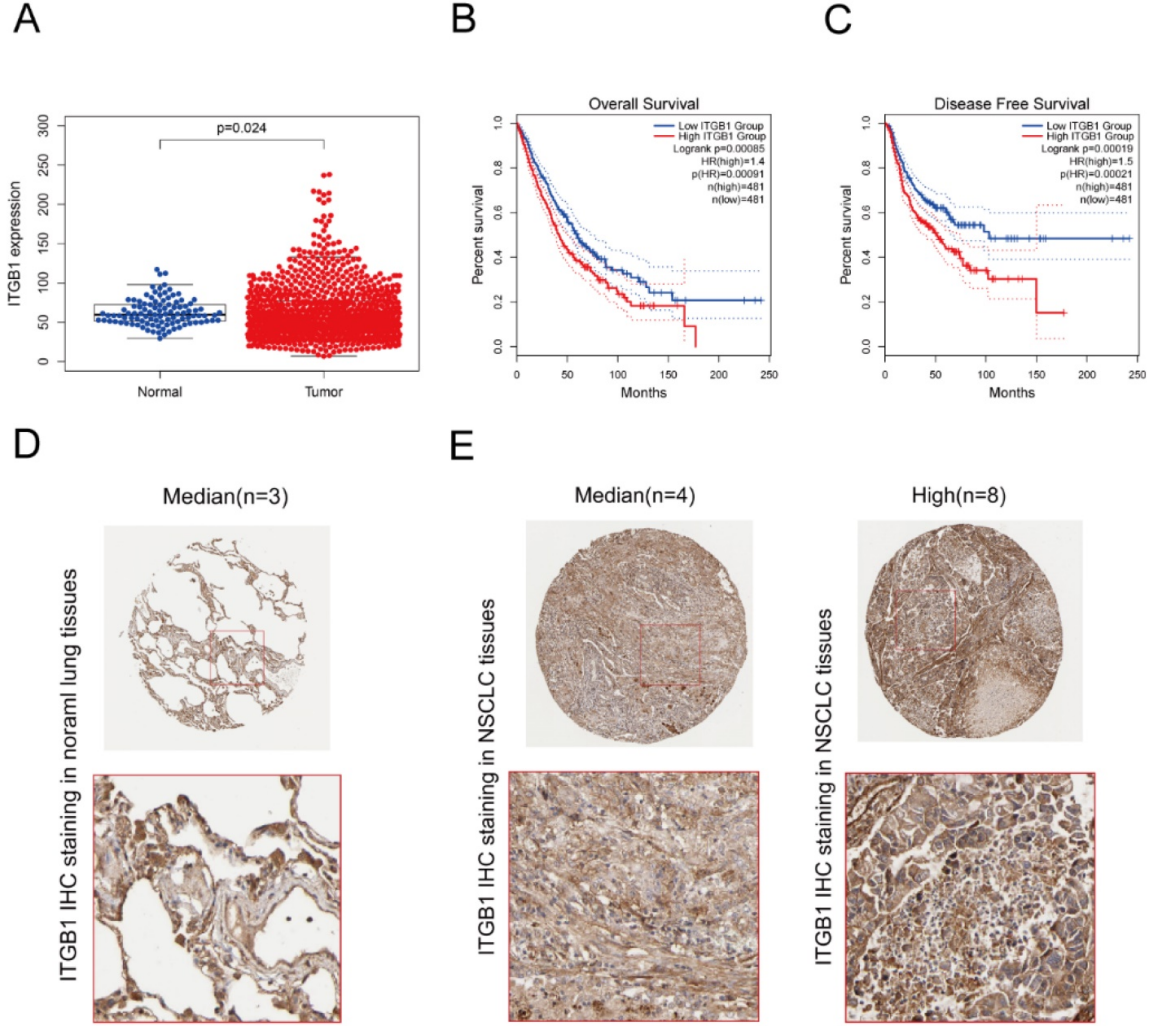
ITGB1 expression negatively correlates with prognosis in patients with NSCLC. A. Analysis of ITGB1 mRNA levels in healthy and NSCLC tissues from the TCGA. B and C. Overall survival and disease-free survival curves stratified by ITGB1 expression for LUAD and LUSC based on data from the GEPIA2 database. D and E. Representative immunohistochemistry images of ITGB1 (antibody clone CAB003434) in healthy lung tissues and NSCLC tissues from the Human Protein Atlas database.

Interrogation of an NSCLC dataset (1,102 cases including 103 normal tissues and 999 tumour tissues) from TCGA revealed that ITGB1 is overexpressed in NSCLC (Fig. 3A). The immunohistochemical staining data retrieved from the HPA database showed that ITGB1 protein expression is higher in NSCLC tissues than in healthy tissues (Fig. 3D and E). ITGB1 expression was also notably associated with clinical stage, T classification, and M classification (Table 1). NSCLC patients with high ITGB1 expression had poorer prognosis than those with low ITGB1 expression (Fig. 3B, C). Univariate analysis revealed that high ITGB1 expression significantly correlated with poor overall survival (HR: 1.431; 95% CI: 1.221-1.667; *P* < 0.01). Other clinicopathologic variables associated with poor survival include advanced stage, T classification, distant metastasis, and lymph nodes (Table 2). After multivariate analysis, ITGB1 remained independently associated with overall survival (HR: 1.345; CI: 1.147-1.576; *P* < 0.01), along with age (Table 2). Thus, ITGB1 expression is a categorical dependent variable associated with poor prognostic clinicopathologic characteristics in patients with NSCLC.

### ITGB1 knockdown restores the radiosensitivity of NSCLC cell lines with intrinsic and acquired radioresistance, whereas ITGB1 up-regulation induces radioresistance

**Figure 4:**
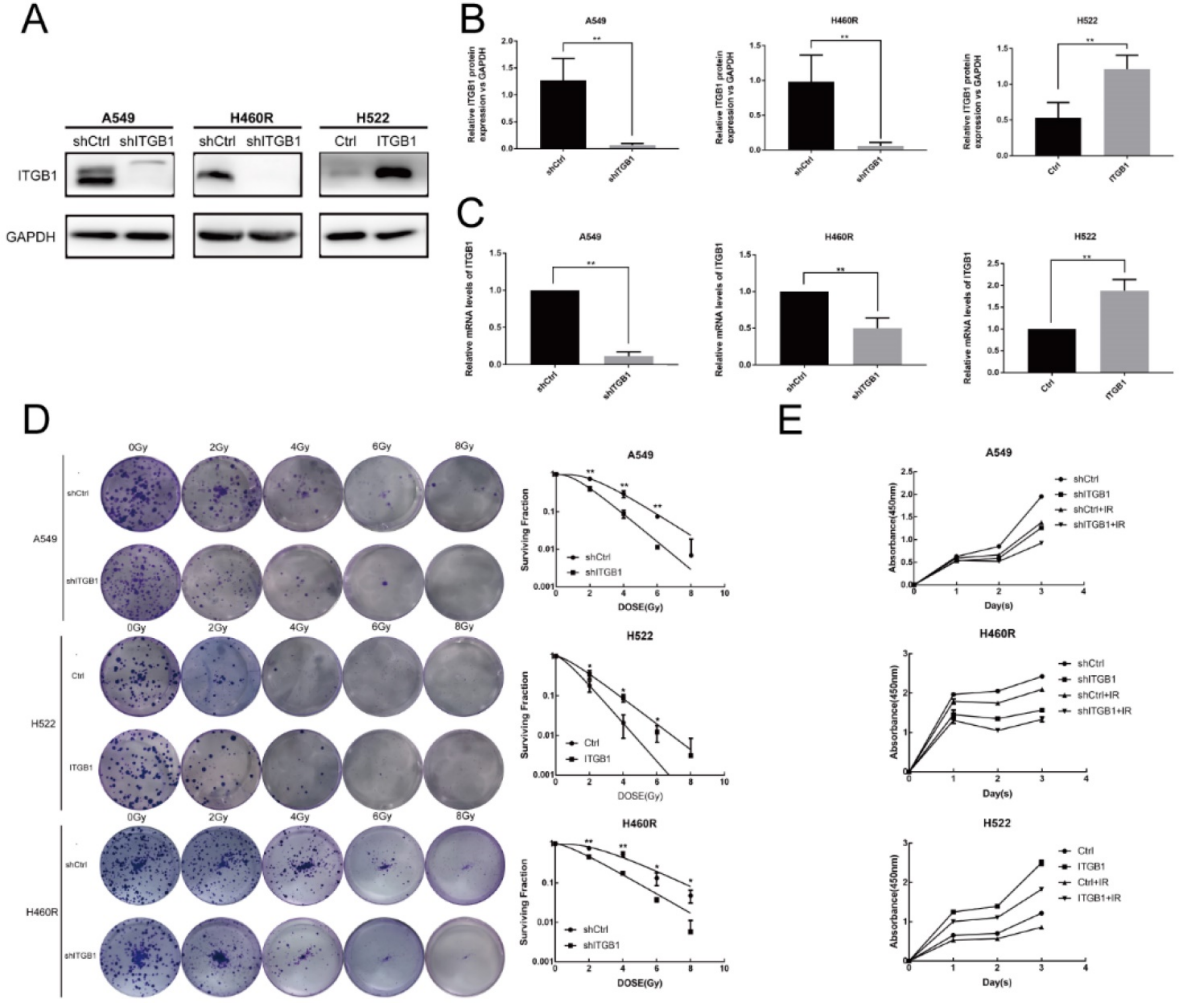
Interfering with ITGB1 expression alter NSCLC cell proliferation and radiosensitivity. A-C. ITGB1 expression was detected by western blot assay and qRT-PCR after lentiviral transduction of A549, H522, and H460R cells. D. Representative photographs of colony formation assays and proportions of surviving A549, H460R, and H522 cells after irradiation with 0, 2, 4, 6, and 8 Gy. E. Cell Counting Kit-8 assays were used to detect the proliferation of A549, H460R, and H522 cells.

To assess the biological function of ITGB1 in NSCLC cell radioresistance, we transduced A549 and H460R cells with a control or shITGB1 vector (A549-shITGB1 and H460R-shITGB1), and up-regulated ITGB1 in H522 cells (H522-ITGB1) using a lentiviral vector. The efficiency of transfection of the cell lines was verified by qRT-PCR and western blotting (Fig. 4A-C). Then, the clonogenic assay was used to analyse the effect of ITGB1 expression on radiosensitivity. A549-shITGB1 and H460R-shITGB1 had a lower survival rate than control-transfected groups post-irradiation. The sensitization enhancement ratio (SER) in the shITGB1 groups were 1.31-fold and 1.24-fold changes from A549 and H460R control groups, respectively (Table 3). Consistent with those findings, H522-ITGB1 cells had a higher survival rate than control-transduced H522 cells (Fig. 4D). The SER in the ITGB1 overexpression group was 0.68-fold change from the H522 control group (Table 3). Results of CCK8 assay exhibited dramatically more proliferation by H522-ITGB1 than by control cells and less proliferation by A549-shITGB1 and H460R-shITGB1 cells than by their respective controls (Fig. 4E). These data indicated that ITGB1 expression was correlated with radiosensitivity, and inhibition of ITGB1 could reverse the intrinsic and acquired radioresistance of NSCLC.

### ITGB1 alters cell cycle status, and blocks irradiation-induced apoptosis

**Figure 5:**
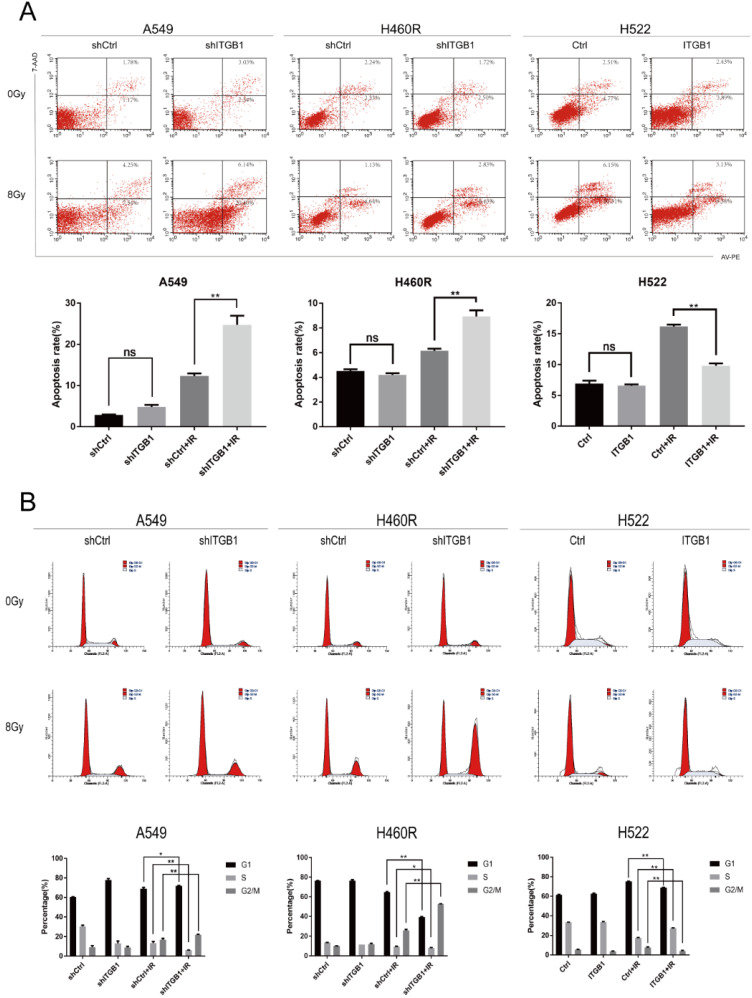
Effect of ITGB1 expression on irradiation-induced apoptosis and cell cycle arrest. A. Flow cytometric apoptosis assay for cells exposed to irradiation (8 Gy). B. Cell cycle analysis after irradiation (8 Gy).

To further observe the effect of ITGB1 in NSCLC cells after IR, we performed flow cytometry to assess cell cycle status. After exposure to ionizing radiation, the proportion of cells in the G2/M phase was higher whereas that in S phase was lower in A549-shITGB1 and H460R-shITGB1 cells than in their negative controls (Fig. 5B). The proportion of H522-ITGB1 cells in the G2/M phase was significantly lower than that of negative control cells, whereas the proportion in the S phase was notably higher (Fig. 5B).

Apoptosis analysis by flow cytometry showed that ITGB1 had no significant influence on NSCLC cell apoptosis in the absence of irradiation. However, 48h after irradiation with 8 Gy, the A549-shITGB1 and H460R-shITGB1 groups had significantly higher proportions of apoptotic cells than the control groups (Fig. 5A). In contrast, irradiation-induced apoptosis occurred at a significantly lower rate in the H522-ITGB1 cells than in the negative control cells (Fig. 5A). These results indicate that ITGB1 inhibition enhances radiation-induced G2/M arrest and apoptosis.

### Targeting ITGB1 expression modulates the DNA damage response

**Figure 6:**
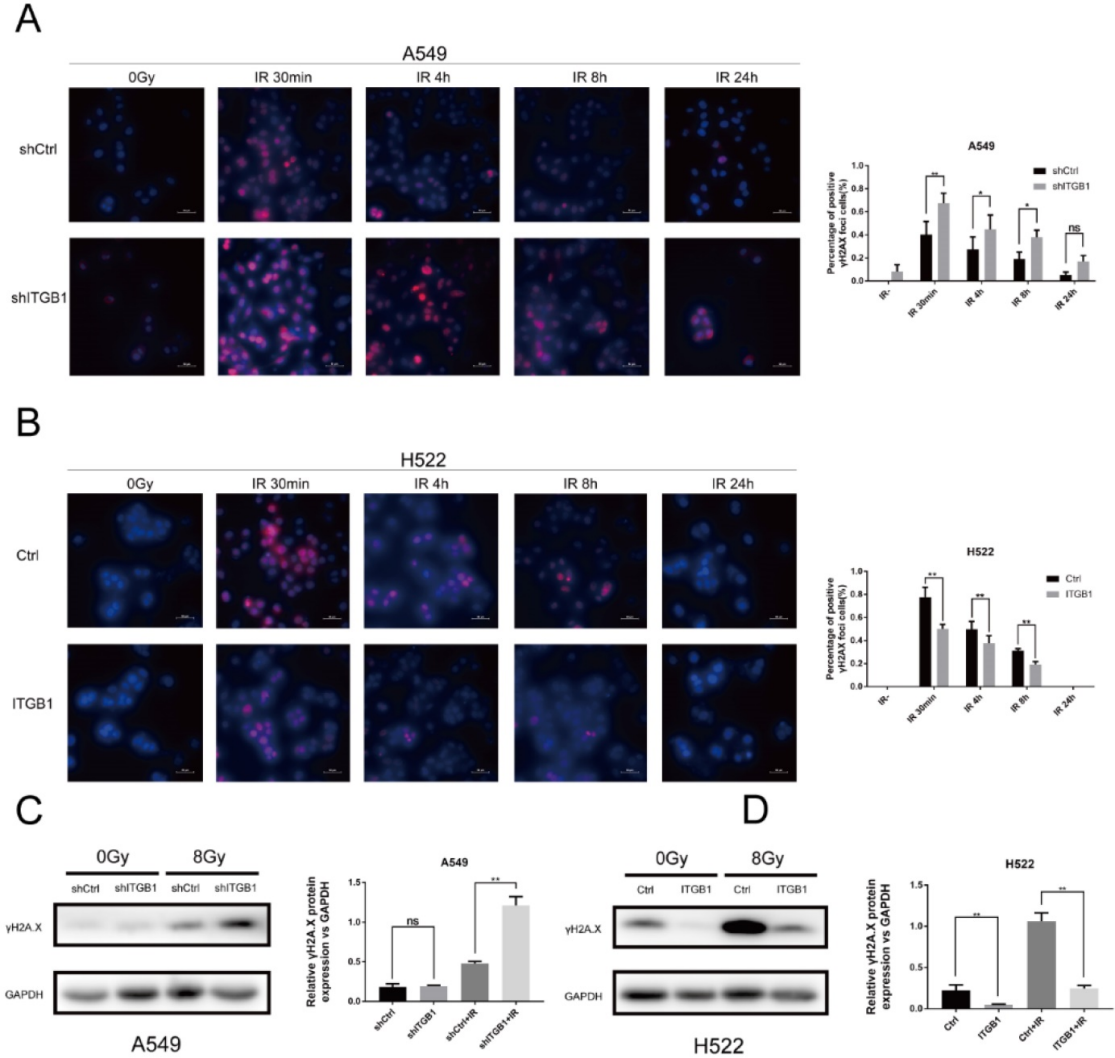
ITGB1 is associated with DNA double-strand breaks (DNA-DSBs) induced by radiation. A and B. Representative images of γH2AX-positive nuclei in shITGB1 and ITGB1 overexpression groups at indicated times following irradiation. γH2AX signal in red, nuclear counterstaining with 4′,6-diamidino-2-phenylindole in blue. Scale bar: 50 µm. γH2AX-positive nuclei were counted in five different fields, each with at least 20 nuclei; the number is shown in relationship to the count in non-irradiated cells. Values represent the average of three independent experiments (right). C and D. Detection of γH2AX protein levels in shITGB1 and ITGB1 overexpression groups treated with or without irradiation (8 Gy).

To further investigate the underlying mechanism associated with ITGB1-induced radioresistance of NSCLC cells, we performed an immunofluorescence assay to detect the expression of γH2AX post-irradiation or in the absence of irradiation. Compared with the non-irradiated cells, the A549-shITGB1 and control cells had significantly more γH2AX-positive nuclei at 30 min post-irradiation (Fig. 6A). However, the H522-ITGB1 cells had fewer nuclei than the control group (Fig. 6B). In both groups, the number of γH2AX-positive nuclei decreased with time post-irradiation and returned to the basal level after 24 h. To confirm these findings, we detected γH2AX protein levels by western blotting. The γH2AX protein levels were markedly increased post-irradiation in both cells. The expression of γH2AX protein was up-regulated in A549-shITGB1 cells (Fig. 6C); however, γH2AX expression was down-regulated in H522-ITGB1 cells in response to irradiation (Fig. 6D).

**Figure 7:**
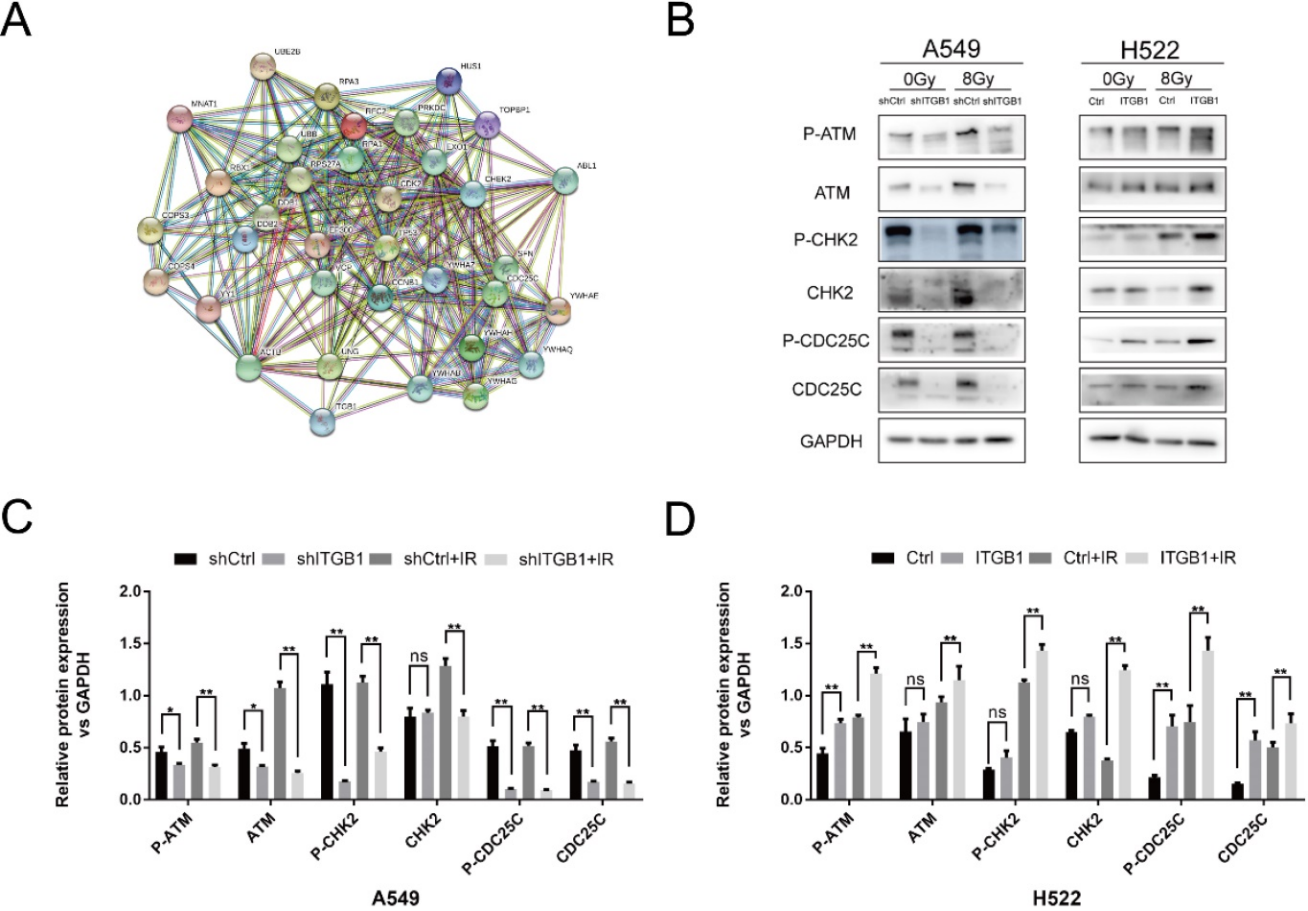
ITGB1 expression correlates with the expression of proteins related to the ATM/CHK2/CDC25c pathway. A. The relationship between ITGB1 expression and that of 34 genes in the DNA-DSB response pathways. B-D. Protein levels of ATM (p-ATM), CHK2 (p-CHK2), and CDC25c (p-CDC25c) were detected by western blotting in shITGB1 and ITGB1 overexpression groups.

To explore the signalling pathway by which ITGB1 performed its functions, we constructed a PPI network for ITGB1 with the genes involved in DNA-DSB repair (Fig. 7A). We found that ITGB1 was potentially related to the ATM/CHK2 axis. We assessed the expression levels of ATM, Chk2, CDC25c, and their phosphorylated variants by western blot. ITGB1 silencing was associated with lower expression of ATM (p-ATM), CHK2 (p-CHK2), and CDC25c (p-CDC25c) in A549 cells, whereas ITGB1 overexpression resulted in higher expression of those proteins in H522 cells (Fig. 7B-D). These results indicate that ITGB1 inhibition suppresses DNA damage repair, thus reversing the intrinsic and acquired radioresistance of NSCLC cells.

### ITGB1 could promote radioresisntance of NSCLC cells by regulating EMT

**Figure 8:**
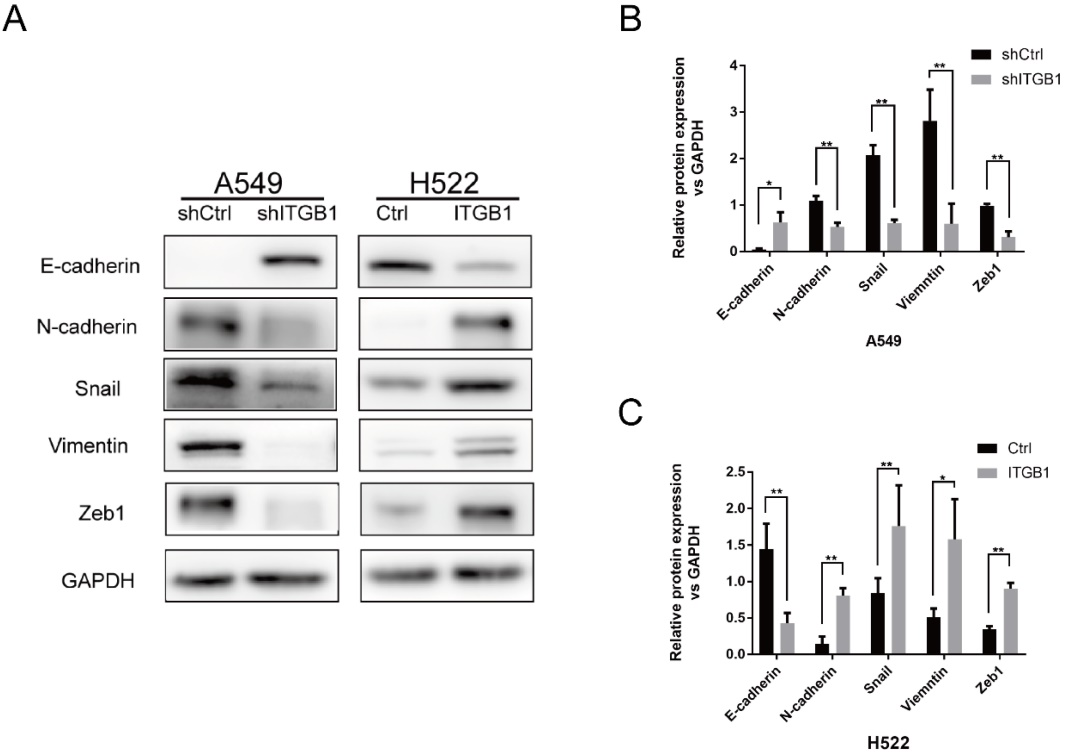
ITGB1 could promote radioresisntance of NSCLC cells by regulating EMT. A-C. Protein levels of E-cadherin, N-cadherin, Snail, vimentin, and Zeb1 were detected by western blotting of cells from shITGB1 and ITGB1 overexpression groups.

To determine whether ITGB1 contributes to NSCLC cell radioresistance by regulating EMT, we analysed the protein levels of epithelial (E-cadherin) and mesenchymal (N-cadherin, Vimentin, Snail, and Zeb1) markers in A549-shITGB1 and H522-ITGB1 cells. Knockdown of ITGB1 was associated with strong inhibition of N-cadherin, vimentin, Snail, and Zeb1 expression and up-regulation of E-cadherin expression in A549 cells. In contrast, ITGB1 overexpression down-regulated the expression of E-cadherin and up-regulated the expression of N-cadherin, vimentin, Snail, and Zeb1 in H522 cells (Fig. 8A-C). These data suggest that ITGB1 regulates radioresistance by inducing the EMT program.

### YAP1 is involved in ITGB1-mediated radioresistance in NSCLC cells

**Figure 9:**
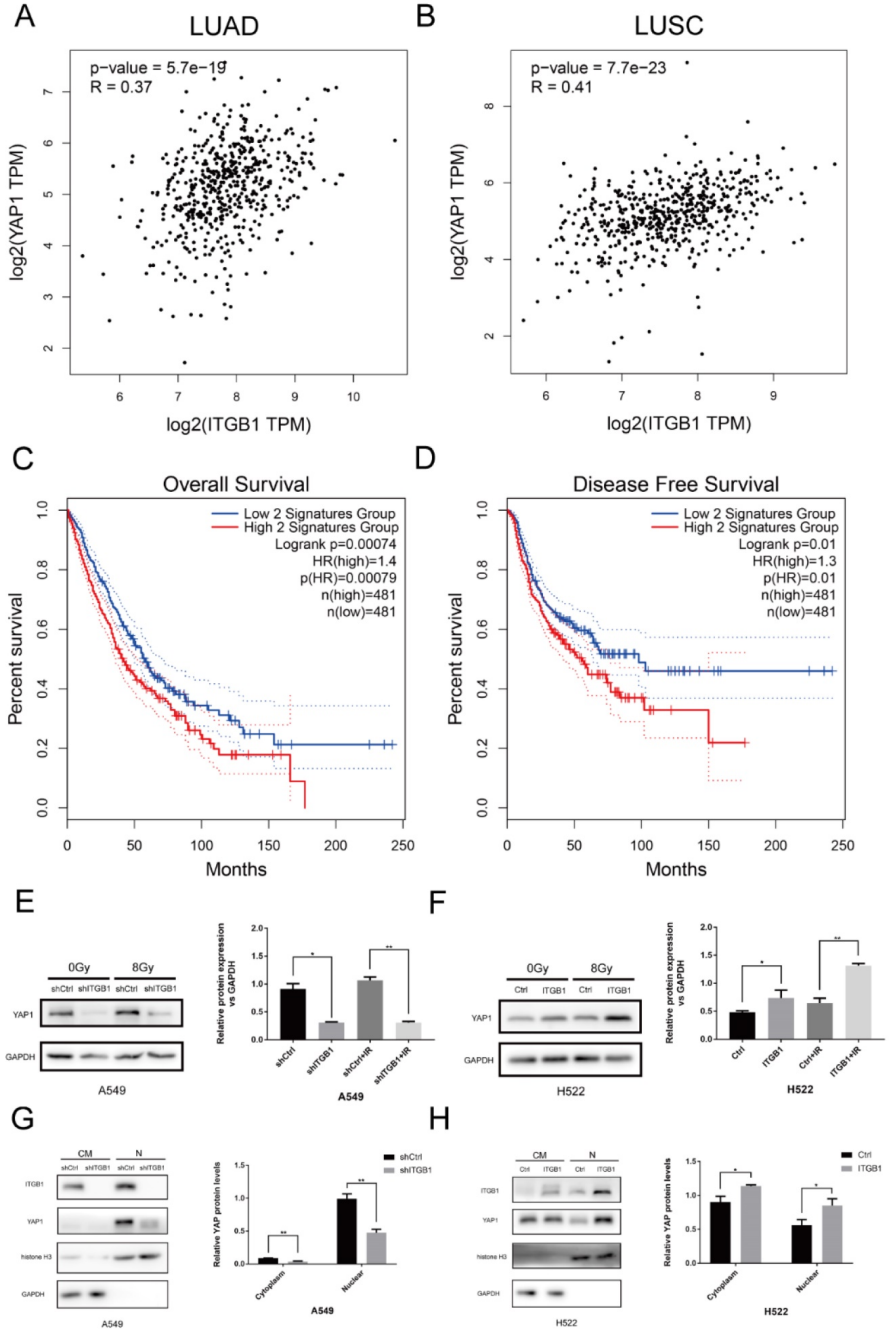
YAP1 is involved in the radioresistance mediated by ITGB1 overexpression in NSCLC. A and B. The correlation between ITGB1 and YAP1 expression in LUAD and LUSC based on data from the GEPIA2 database. C and D. Kaplan-Meier curves depict the probability of overall survival and disease-free survival based on the expression of ITGB1 and YAP1. Data from patients with LUAD and LUSC retrieved from the GEPIA2 database. E and F. Western blot analysis of total YAP1 protein levels. G and H. Nuclear and cytoplasmic YAP1 protein levels in shITGB1-treated and ITGB1-overexpressing cells.

We investigated the correlation between ITGB1 and YAP1 expression in LUAD, LUSC, and healthy tissue samples using GEPIA2 (Fig. 9A and B). ITGB1 expression positively correlated with YAP1 expression in LUAD (Cor = 0.37, *P* < 0.01) and LUSC (Cor = 0.41, *P* < 0.01) samples. Furthermore, survival analyses of NSCLC patients in GEPIA2 showed that high levels of these two genes predicted significantly shorter overall survival and worse disease-free survival (Fig. 9C and D). To verify the role of YAP1 in radioresistance, we targeted YAP1 with a specific siRNA in H460R cells; YAP1 down-regulation more significantly promoted apoptosis post-irradiation compared to transfection with control siRNA (Fig. S1). Western blot analysis showed that YAP1 was down-regulated when ITGB1 was knocked down in A549 cells, whereas YAP1 expression was up-regulated in the H522-ITGB1 group (Fig. 9E and F). Western blotting showed that ITGB1 knockdown significantly impaired YAP1 nuclear localization in A549 cells (Fig. 9G), whereas overexpression was associated with significantly increased nuclear YAP1 localization in H522-ITGB1 cells (Fig. 9H). These results indicate that YAP1 may be involved in the ITGB1-mediated radioresistance of NSCLC cells.

## Discussion

We used RNA sequencing to investigate the differentially expressed genes in radioresistant H460R and radiosensitive H460 cells. ECM-receptor interaction, focal adhesion, and cell adhesion molecule signalling pathways, which essentially contribute to tumour cell resistance to radiation and chemotherapy, were found to be enriched by KEGG analysis. We created a heatmap of genes that were enriched in these pathways. ITGB1 was chosen for further study based on its association with the tumour ECM.

ITGB1, which coordinates proliferation, migration, invasion, adhesion, and inflammation, has recently been implicated in the therapeutic resistance of multiple solid cancers 11, 14, 18, 54. In HNSCC, ITGB1 inhibition enhances radiosensitivity and impairs DNA repair, resulting in increased residual DNA damage levels 25, 55. ITGB1 inhibition suppresses the invasion of laryngeal cancer cells and reduces their radioresistance by targeting CD147 56. In pancreatic cancer, ITGB1 promotes gemcitabine resistance via CDC42 activation of PI3K p110β signalling 23. ITGB1 overexpression is also associated with resistance to the tyrosine kinase inhibitor gefitinib in NSCLC 24. In the present study, we observed significantly higher levels of ITGB1 in NSCLC cells with acquired radioresistance than in radiosensitive cells. Moreover, H522, which has a low ITGB1 expression, was more sensitive to radiation than A549, which has high ITGB1 expression. Bioinformatic analysis using high-throughput RNA sequencing data from TCGA demonstrated that increased expression of ITGB1 in NSCLC was associated with advanced clinical pathologic characteristics, shorter survival, and poor prognosis. As such, ITGB1 may be associated with radiation-resistant NSCLC as a biomarker and therapeutic target.

To clarify its role in radioresistance, we manipulated the expression of ITGB1 in A549, H522, and radioresistant H460R cells. Both silencing and overexpression of ITGB1 revealed that it was associated with clonogenic survival rate, cell viability, and resistance to radiation-induced apoptosis. Our findings suggest a correlation between ITGB1 expression and NSCLC cell radioresistance, so we explored the possible mechanisms of ITGB1-induced radioresistance.

Irradiation induces DNA-DSBs, and the ability to repair DNA-DSBs is closely associated with sensitivity to radiotherapy 47, 48. γH2AX, an early marker of DNA-DSBs, is positively associated with radiosensitivity. Our study demonstrated that the number of γH2AX-positive nuclei in A549-shITGB1 cells was significantly higher than that in control cells post-irradiation. Moreover, H522-ITGB1 cells exhibited fewer γH2AX-positive nuclei, indicating enhanced DNA-DSB repair upon overexpression of ITGB1. Irradiation-induced DNA-DSBs are primarily repaired by nonhomologous end-joining and homologous recombination 57. We investigated the role of ITGB1 in these two repair pathways using the PathCards database, which revealed that multiple key proteins that participate in DNA-DSB repair pathways are directly or indirectly connected with ITGB1. One of those genes, CHK2 (CHEK2) is a key component of the DNA damage response. Following DNA damage, CHK2 is phosphorylated by ATM at Thr68 site 58. In human cells, activation of ATM/CHK2 signalling leads to phosphorylation of more than 20 proteins that induce an appropriate cellular response; depending on the extent of damage, the cell type, and other factors, the response may be cell cycle checkpoint activation, induction of apoptosis or senescence, DNA repair, or tolerance to the damage 59. Lin et al. found that ATM phosphorylation and CHK2 expression were significantly higher in GBM-R2M2 than GBM-Par cells, which resulted in more efficient DNA repair, cell motility, and survival after irradiation 60. CHK2 is regarded as a tumour suppressor due to its role in apoptosis regulation with or without the involvement of ATM; upon phosphorylation, activated CHK2 phosphorylates the tumour suppressor gene P53 and multiple CDC25 molecules that negatively regulate kinase activation in a cyclin-dependent manner, which may provide insight into the potential mechanism of ITGB1-mediated radioresistance 61. Our results indicated that ITGB1 expression affects the expression of proteins related to the ATM/CHK2/CDC25c pathway.

The EMT phenotype, together with cancer stem cell properties, has been implicated in increased resistance to radiotherapy 62. The EMT markers were analyzed in Human Protein Atlas database to examine their protein levels in NSCLC. Their implication in patient survival was also analyzed (Fig. S2-6). The results indicated that each of EMT markers was not independent prognostic factor. Interestingly, co-expression of the four mesenchymal markers (N-cadherin, Vimentin, Snail, and Zeb1) predicted significantly poor overall survival and worse disease-free survival (Fig. S7). They might cooperate with each other to affect the occurrence and development of tumors and the resistance to therapy. Previously study showed that E-cadherin loss and Mesenchymal conversion were associated with radio-resistance in human tumor cells 63. We showed that blocking ITGB1 down-regulated the expression of mesenchymal genes and the EMT-promoting gene ZEB1 while up-regulating E-cadherin protein expression. Moreover, ITGB1 overexpression caused the opposite results, suggesting that enhanced ITGB1 expression leads to EMT in NSCLC cells, thereby increasing their resistance to irradiation. The transcriptional coactivator YAP1 is involved in EMT regulation, and its expression in NSCLC is associated with cancer progression and poor prognosis 64, 65. In our study, depletion of YAP1 enhanced H460R radiosensitivity. YAP1 acts as a nuclear sensor for mechanotransduction, which can be induced by stiffness of the ECM 66. We found that ITGB1 expression positively correlated with YAP1 expression and the two-gene expression signature correlated with the survival of patients with NSCLC. Our study revealed that ITGB1 silencing reduced total YAP1 expression and inhibited YAP1 nuclear localization. Overexpression of ITGB1 enhanced YAP1 expression and restored its nuclear localization, thus confirming our hypothesis that ITGB1 promotes EMT and radioresistance by targeting YAP1.

In conclusion, our results suggest that ITGB1 promotes EMT by targeting YAP1 and influences the DNA damage response by affecting cell cycle checkpoints, apoptosis, and ATM/CHK2 signalling, whereas inhibiting ITGB1 reverses those effects and improves NSCLC radiosensitivity. Our findings support the use of ITGB1 as a biomarker for prognosis and as a novel therapeutic target for increasing NSCLC radiosensitivity.

## Supplementary Material

Supplementary figures and tables.

## Figures and Tables

**Table 1:**
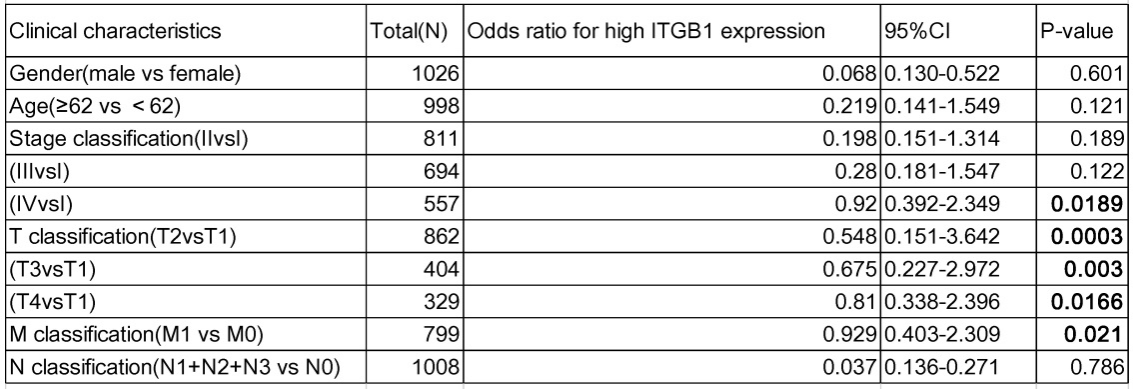
The association between ITGB1 expression and clinic-pathological characteristics of NSCLC patients

CI: confidence interval; bold values indicate *P*<0.05.

**Table 2:**
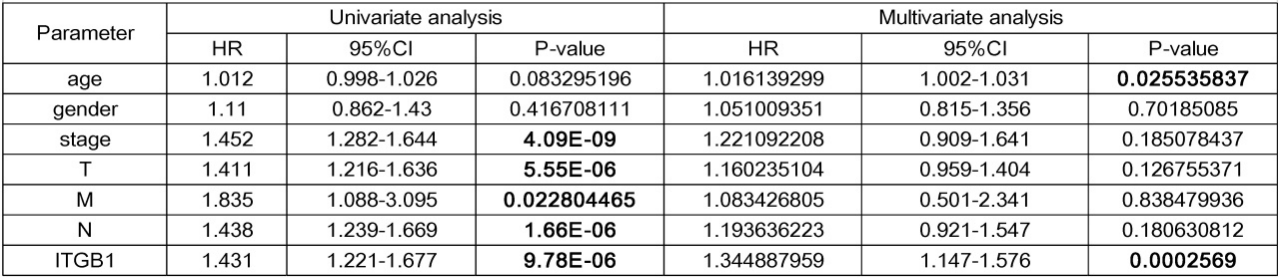
Univariate and multivariate analyses of the relationship between ITGB1 expression and overall survival among NSCLC patients

**Table 3:**
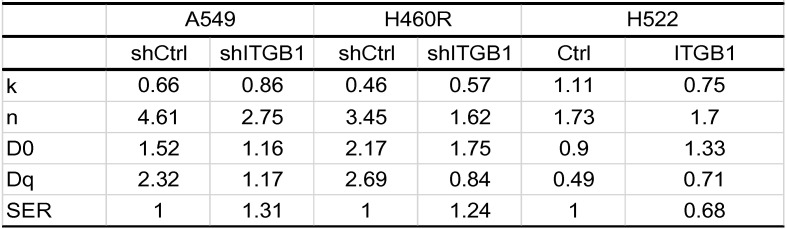
Radiation biologic parameter of NSCLC cells exposed to radiation
