# Supplementary material for: ITGB1 enhances the Radioresistance of human Non-small Cell Lung Cancer Cells by modulating the DNA damage response and YAP1-induced Epithelial-mesenchymal Transition
Source: Int J Biol Sci. 2021 Jan 18;17(2):635–50. doi: 10.7150/ijbs.52319 (PMC7893583; doi:10.7150/ijbs.52319)
Supplement: Supplementary file 1 — Supplementary figures and tables. [file ijbsv17p0635s1.pdf]

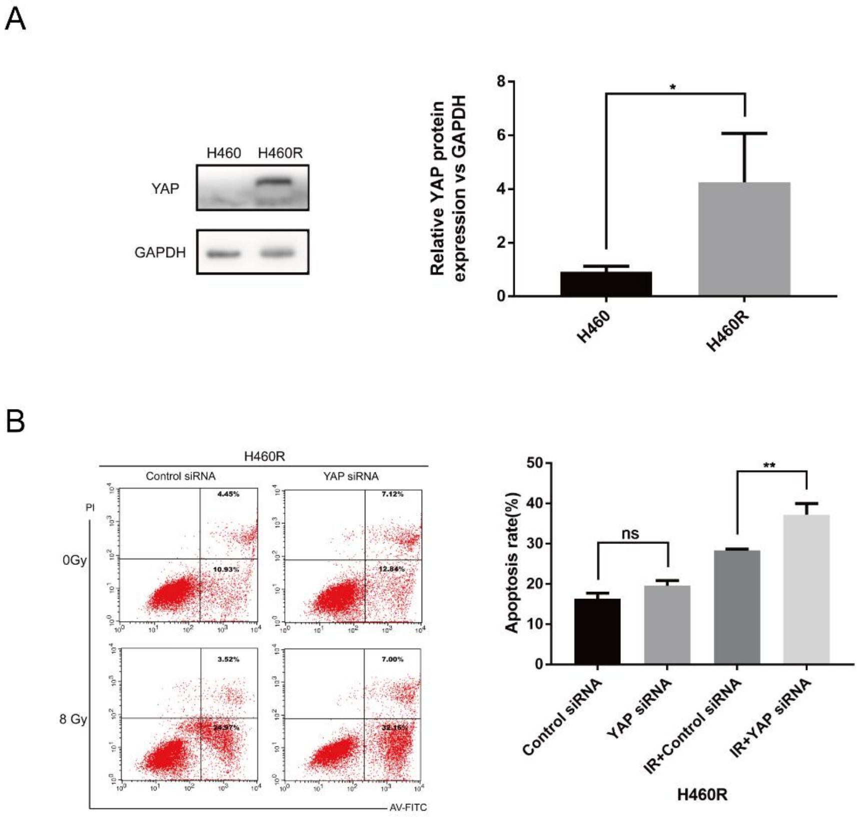

S1. Inhibition of *YAP1* sensitized cells to radiation-induced apoptosis. A. The *YAP1* protein level was detected by western blot in H460R and H460 cells. B. Apoptosis assay for *YAP1* siRNA-treated cells and control cells following irradiation (8 Gy).

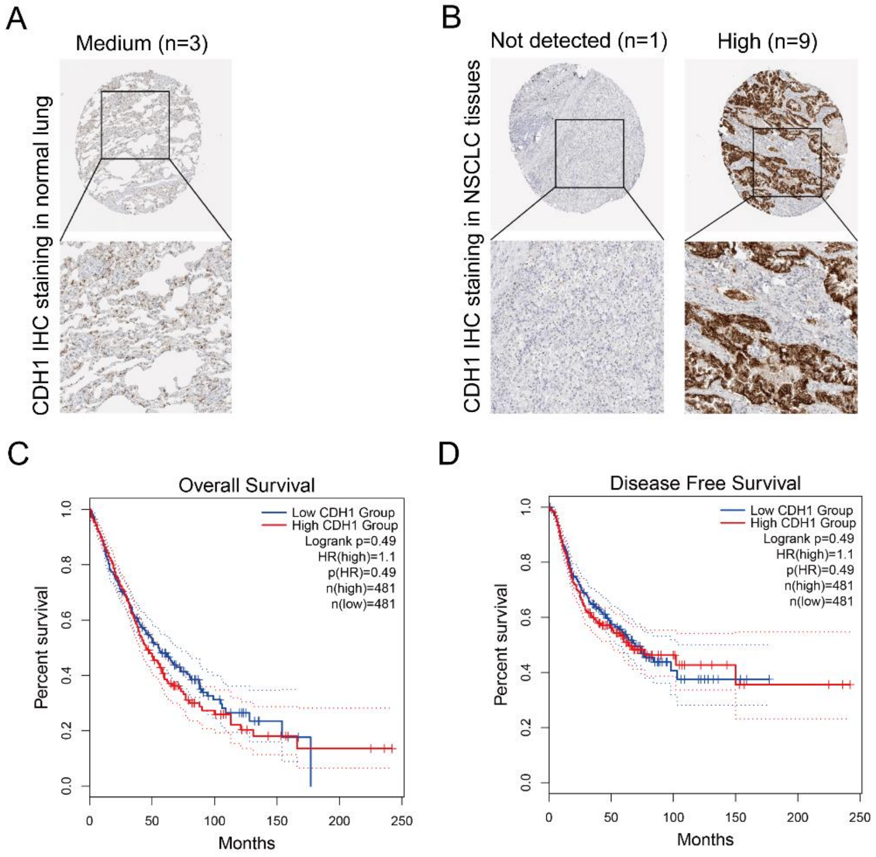

S2. A and B. Representative immunohistochemistry images of E-cadherin (antibody clone CAB072857) in healthy lung tissues and NSCLC tissues from the Human Protein Atlas database. C and D. Overall survival and disease-free survival curves stratified by E-cadherin expression for LUAD and LUSC based on data from the GEPIA2 database.

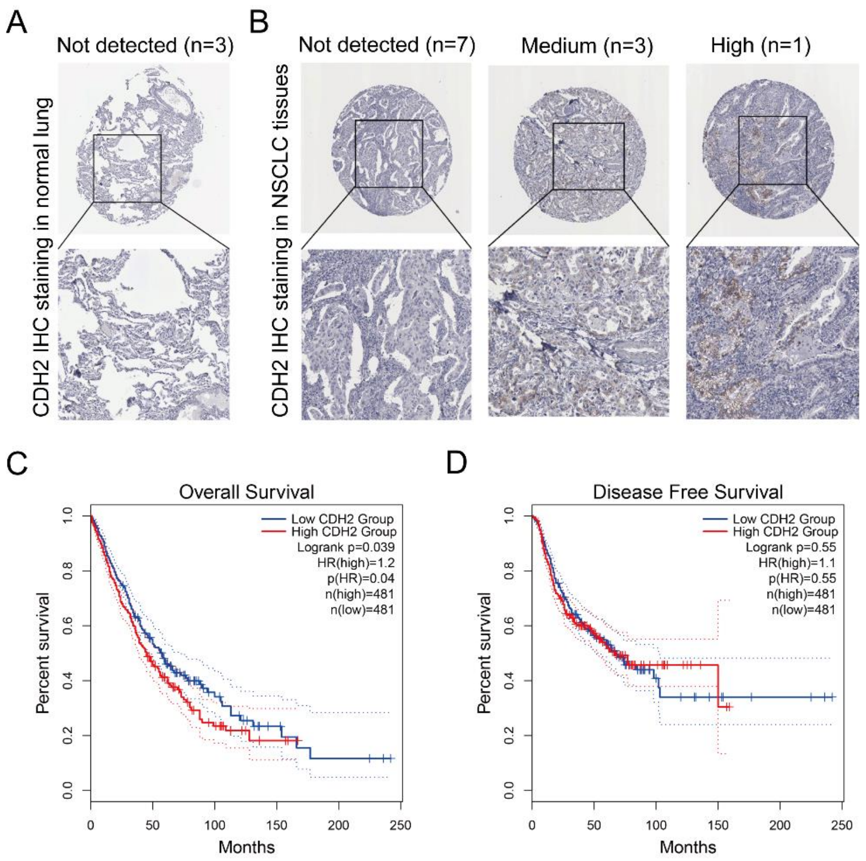

S3. A and B. Representative immunohistochemistry images of N-cadherin (antibody clone CAB000141) in healthy lung tissues and NSCLC tissues from the Human Protein Atlas database. C and D. Overall survival and disease-free survival curves stratified by N-cadherin expression for LUAD and LUSC based on data from the GEPIA2 database.

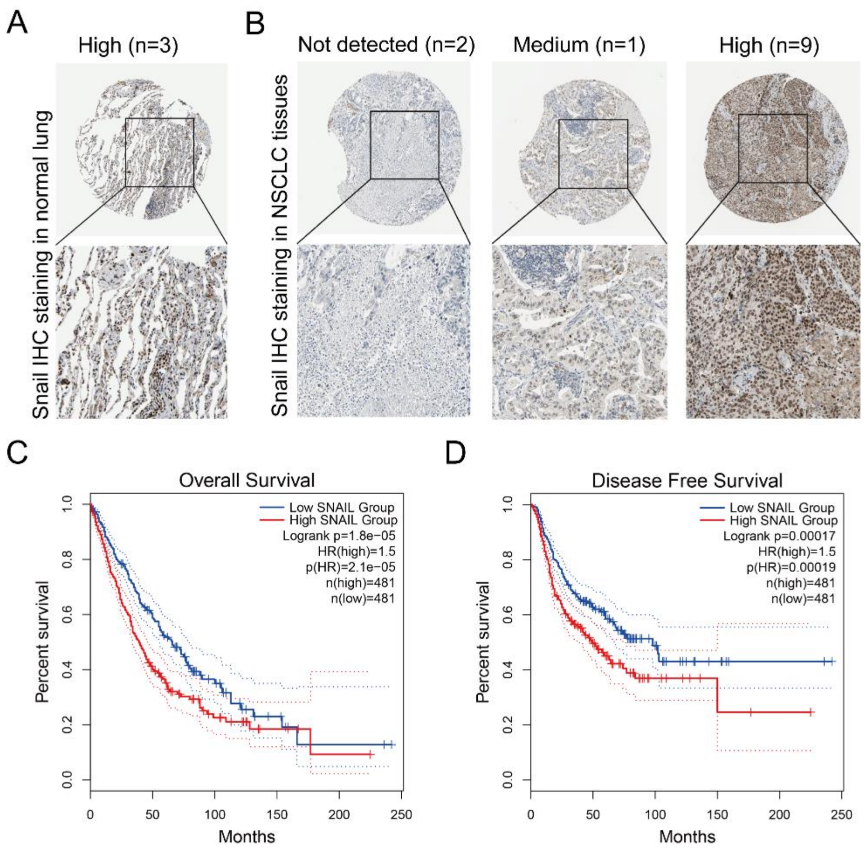

S4. A and B. Representative immunohistochemistry images of Snail (antibody clone CAB005883) in healthy lung tissues and NSCLC tissues from the Human Protein Atlas database. C and D. Overall survival and disease-free survival curves stratified by Snail expression for LUAD and LUSC based on data from the GEPIA2 database.

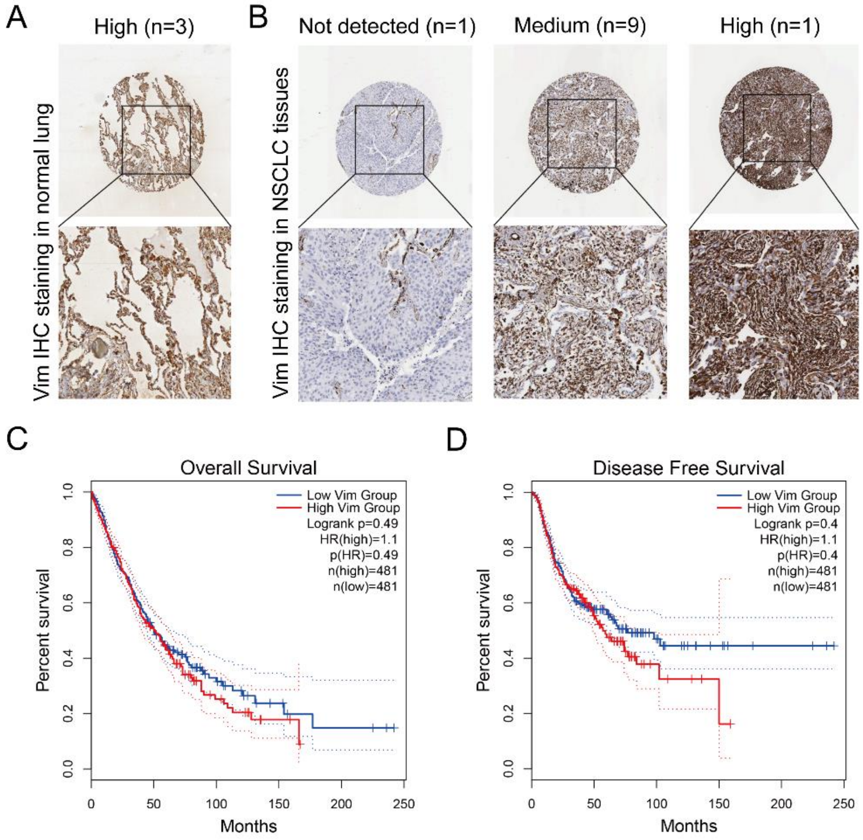

S5. A and B. Representative immunohistochemistry images of Vimentin (antibody clone CAB000080) in healthy lung tissues and NSCLC tissues from the Human Protein Atlas database. C and D. Overall survival and disease-free survival curves stratified by Vimentin expression for LUAD and LUSC based on data from the GEPIA2 database.

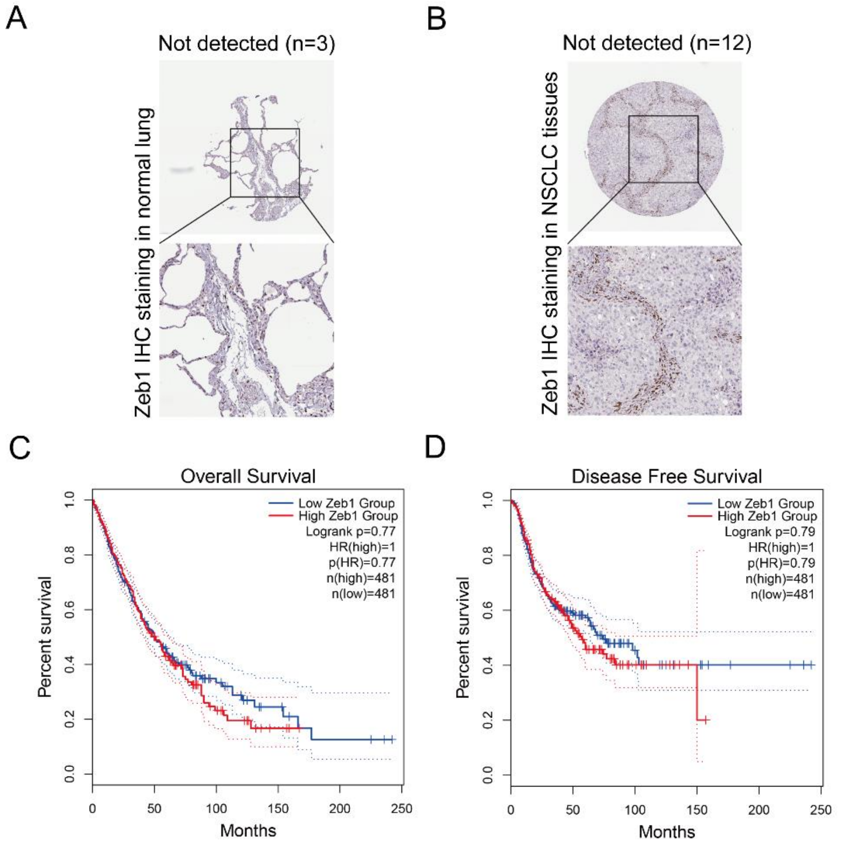

S6. A and B. Representative immunohistochemistry images of Zeb1 (antibody clone CAB058686) in healthy lung tissues and NSCLC tissues from the Human Protein Atlas database. C and D. Overall survival and disease-free survival curves stratified by Zeb1 expression for LUAD and LUSC based on data from the GEPIA2 database.

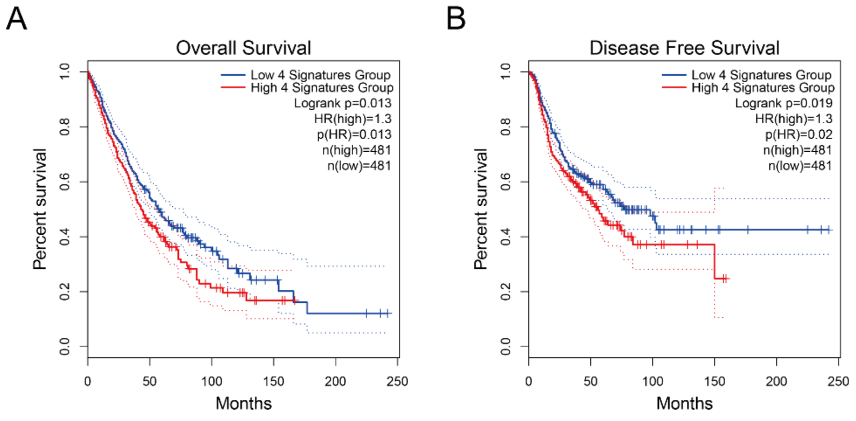

S7. A and B. Kaplan–Meier curves depict the probability of overall survival and disease-free survival based on the expression of CDH2, Snail, Vimentin and Zeb1. Data from patients with LUAD and LUSC retrieved from the GEPIA2 database.

Table S1

id	log2(FC)	Pvalue	FDR	significant	Symbol
ENST00000005558	-1.6763	5.38E-06	0.001308	yes	IFRD1
ENST00000012134	8.16323	0.000519	0.043957	yes	HIVEP2
ENST00000014914	1.276526	8.60E-07	0.000277	yes	GPRC5A
ENST00000040738	2.005806	9.26E-11	1.02E-07	yes	BOD1L1
ENST00000199940	-1.35816	0.000508	0.043128	yes	MAP2
ENST00000216281	-1.04904	0.00048	0.041308	yes	HSP90AA1
ENST00000218388	2.063192	2.55E-12	4.16E-09	yes	TIMP1
ENST00000220584	-1.4803	0.000327	0.031088	yes	FDFT1
ENST00000222399	1.50885	1.70E-05	0.003107	yes	LAMB1
ENST00000222543	1.347111	6.03E-06	0.001425	yes	TFPI2
ENST00000223095	1.742042	4.54E-05	0.00682	yes	SERPINE1
ENST00000224652	10.07236	2.31E-05	0.003979	yes	ATE1
ENST00000226253	-2.26356	0.000178	0.020051	yes	ALDOC
ENST00000232892	2.151663	1.70E-06	0.000487	yes	AADAC
ENST00000234179	-10.7841	2.72E-06	0.000721	yes	PRKD3
ENST00000235307	1.532578	0.000387	0.035193	yes	C1orf21
ENST00000236957	-1.23008	0.000604	0.04908	yes	EEF1B2
ENST00000238682	-2.40523	9.29E-05	0.011994	yes	TGFB3
ENST00000242607	-3.01042	1.26E-07	5.18E-05	yes	HVCN1
ENST00000243213	4.595703	7.28E-09	4.69E-06	yes	IL13RA2
ENST00000243349	3.655884	1.51E-08	8.36E-06	yes	ACVR1C
ENST00000243924	-4.34162	1.14E-11	1.58E-08	yes	PI3
ENST00000245479	1.605762	7.50E-05	0.010204	yes	SOX9
ENST00000245907	-4.04313	3.89E-40	2.66E-35	yes	C3
ENST00000246672	1.389333	0.000216	0.022851	yes	NR1D1
ENST00000248437	-1.27453	0.000276	0.027533	yes	TUBA4A
ENST00000250340	-2.93842	8.27E-07	0.000268	yes	CLEC11A
ENST00000252804	-9.45806	4.45E-09	2.98E-06	yes	PXDN
ENST00000252809	1.783102	9.73E-08	4.08E-05	yes	GDF15
ENST00000252999	1.052318	8.54E-05	0.011277	yes	LAMA5
ENST00000256015	1.566064	1.57E-06	0.000461	yes	BTG1
ENST00000256442	-1.26691	0.000161	0.018693	yes	CCNB1
ENST00000256737	-8.74819	1.46E-06	0.000432	yes	ANO3
ENST00000256759	2.301084	3.73E-05	0.005902	yes	FST
ENST00000256951	1.364099	0.000106	0.01327	yes	EMP1
ENST00000257261	-2.15095	2.15E-07	8.12E-05	yes	FADS2
ENST00000257527	3.728798	1.22E-16	4.38E-13	yes	ADAM19
ENST00000257770	3.400923	1.36E-27	3.09E-23	yes	NT5E
ENST00000258349	8.667703	7.62E-05	0.010302	yes	RC3H1
ENST00000258405	1.014668	0.000136	0.01632	yes	SERPINE2
ENST00000259605	9.557145	0.000154	0.018018	yes	RNF38
ENST00000259818	-1.74045	6.39E-05	0.009005	yes	TUBB2B
ENST00000261173	10.93909	0.000102	0.012891	yes	ATP2B1
ENST00000261233	-3.78427	1.21E-05	0.002459	yes	IRAK3
ENST00000261435	1.261423	0.000427	0.037655	yes	N4BP2
ENST00000261479	-1.15531	0.000226	0.023626	yes	PSMA6
ENST00000261654	1.022192	0.00033	0.0313	yes	ADGRD1
ENST00000262030	-1.11263	0.000211	0.022522	yes	ATP5F1B
ENST00000262067	-1.44557	0.000506	0.043128	yes	TSPAN13
ENST00000262244	8.50515	7.25E-06	0.001657	yes	MOB3B
ENST00000262304	1.170019	9.36E-05	0.011996	yes	PKD1
ENST00000262318	1.132968	0.000536	0.044832	yes	CLCN7
ENST00000262982	-1.06578	0.000274	0.027412	yes	CSE1L
ENST00000263826	1.988945	6.75E-07	0.000226	yes	AKT3
ENST00000264071	-1.99866	2.71E-06	0.000721	yes	TUBB4A
ENST00000264156	-1.3326	2.48E-05	0.004163	yes	MCM6

ENST00000264463	-1.46969	1.57E-05	0.002972	yes	CDH10
ENST00000264596	-1.1921	0.000244	0.025108	yes	NEIL3
ENST00000264605	1.906187	1.20E-08	6.95E-06	yes	MLPH
ENST00000265097	-1.19951	0.000374	0.034221	yes	THOC3
ENST00000265361	2.415037	1.02E-07	4.26E-05	yes	SEMA3C
ENST00000265362	1.144966	1.52E-05	0.002904	yes	SEMA3A
ENST00000265678	2.169127	1.35E-05	0.002657	yes	RPS6KA2
ENST00000265724	2.224373	4.61E-05	0.00688	yes	ABCB1
ENST00000265843	1.851749	5.98E-06	0.001417	yes	EXPH5
ENST00000266037	2.054613	3.21E-05	0.00526	yes	DOCK3
ENST00000266070	-11.5825	1.69E-25	1.93E-21	yes	DIDO1
ENST00000266682	1.131783	6.80E-05	0.009442	yes	SLC6A15
ENST00000267079	9.076816	8.66E-06	0.001903	yes	MAP3K12
ENST00000268613	1.184692	2.41E-05	0.004097	yes	CDH13
ENST00000269243	-1.69037	1.91E-07	7.40E-05	yes	MYH10
ENST00000270142	-1.06309	0.000599	0.048685	yes	SOD1
ENST00000270458	-7.54432	0.000183	0.020344	yes	CACNG8
ENST00000271583	10.01309	0.000294	0.028991	yes	TOR1AIP1
ENST00000273990	-1.66935	6.24E-06	0.001455	yes	DDIT4L
ENST00000276659	-3.09782	3.01E-05	0.004967	yes	RSPO2
ENST00000276699	-4.03002	3.87E-05	0.006082	yes	FAM83A
ENST00000278840	-2.61875	1.69E-11	2.22E-08	yes	FADS2
ENST00000280591	10.41996	0.000371	0.034043	yes	TRNT1
ENST00000281092	1.764961	6.89E-05	0.009548	yes	FER
ENST00000281821	1.314612	3.48E-05	0.005557	yes	EPHA4
ENST00000282050	-1.03992	0.000346	0.032334	yes	ATP5F1A
ENST00000282588	1.504043	0.000206	0.022014	yes	ITGA1
ENST00000283871	-2.80873	1.08E-05	0.002275	yes	HGD
ENST00000284037	1.319851	0.000299	0.029367	yes	ERBIN
ENST00000284273	1.79187	1.10E-06	0.000343	yes	UBASH3B
ENST00000284894	-2.49632	8.06E-06	0.001788	yes	NCAM2
ENST00000285599	1.273638	5.20E-05	0.007605	yes	MAN2B2
ENST00000286627	9.78136	8.66E-06	0.001903	yes	KCNMA1
ENST00000286824	12.91687	7.96E-06	0.001771	yes	TSPAN7
ENST00000288207	-1.23476	0.000263	0.026662	yes	CCNB2
ENST00000288381	7.78136	0.000475	0.040981	yes	TMEM164
ENST00000289004	-3.81511	2.12E-05	0.00371	yes	HPD
ENST00000290271	1.652077	0.000345	0.03232	yes	STC1
ENST00000295190	-1.49757	2.17E-05	0.00378	yes	SLC16A14
ENST00000295400	3.528379	9.05E-05	0.011758	yes	TGFA
ENST00000295927	2.726284	2.59E-10	2.49E-07	yes	PTX3
ENST00000295956	10.86625	1.93E-07	7.47E-05	yes	FLNB
ENST00000296585	2.228479	5.33E-11	6.38E-08	yes	ITGA2
ENST00000296677	3.878777	1.75E-05	0.003177	yes	F2RL1
ENST00000297438	-1.79156	6.74E-05	0.009392	yes	OSGIN2
ENST00000297564	-12.3826	1.60E-05	0.003001	yes	COX6C
ENST00000297625	-3.36457	5.80E-06	0.001386	yes	MYORG
ENST00000297848	-1.30918	0.00019	0.020793	yes	COL14A1
ENST00000297991	-3.8909	8.67E-11	9.71E-08	yes	AQP3
ENST00000298892	1.927504	5.42E-06	0.001312	yes	CAPRIN2
ENST00000298912	1.968912	0.000598	0.04867	yes	CLMN
ENST00000299502	-1.03929	0.000401	0.035962	yes	SERPINB2
ENST00000300006	1.115927	0.000583	0.047723	yes	C16orf45
ENST00000300403	-1.00283	0.000419	0.037117	yes	TPX2
ENST00000301071	-1.84742	9.03E-08	3.88E-05	yes	TUBA1A
ENST00000301178	1.762113	3.42E-05	0.005496	yes	AXL
ENST00000301634	-1.25395	0.000621	0.049968	yes	TK1
ENST00000301691	-5.61872	2.35E-14	5.79E-11	yes	SOST

ENST00000301732	1.157284	0.000201	0.021702	yes	ABCA3
ENST00000301904	-1.62209	0.000333	0.031528	yes	SCARA3
ENST00000301905	-1.12833	0.00053	0.044757	yes	PBK
ENST00000303115	3.411426	0.000389	0.035313	yes	IL7R
ENST00000303910	-1.20226	7.79E-05	0.010437	yes	HIST1H2AE
ENST00000304385	2.034859	8.46E-05	0.011203	yes	TMEM154
ENST00000304567	-1.35764	9.12E-07	0.00029	yes	RRM2
ENST00000305414	-2.62161	1.66E-13	3.43E-10	yes	FGF13
ENST00000307365	-1.0959	0.000276	0.027533	yes	DDIT4
ENST00000309052	-8.80305	2.33E-05	0.004005	yes	LCLAT1
ENST00000309083	-1.13638	0.000423	0.037396	yes	PTMS
ENST00000309851	-1.25916	0.000203	0.021775	yes	GSTM1
ENST00000310032	-9.82972	1.58E-05	0.002976	yes	TSPAN17
ENST00000310397	2.776622	4.26E-06	0.001066	yes	SNED1
ENST00000310454	-1.45464	0.000444	0.038945	yes	GPD2
ENST00000310455	-1.39468	0.000283	0.028179	yes	PPP1R3B
ENST00000310624	-5.35364	1.40E-09	9.93E-07	yes	NEFH
ENST00000310931	-1.29129	1.24E-05	0.0025	yes	HDLBP
ENST00000311734	10.98252	6.72E-14	1.53E-10	yes	IL1RL1
ENST00000314754	2.071497	2.33E-10	2.28E-07	yes	CD55
ENST00000315366	1.698491	2.45E-05	0.004144	yes	ARSJ
ENST00000315930	-2.50596	1.21E-11	1.66E-08	yes	FGF13
ENST00000318024	-3.85354	4.41E-07	0.000158	yes	USH1C
ENST00000318160	-1.63523	1.30E-06	0.000393	yes	GREM2
ENST00000319211	2.148826	8.89E-07	0.000285	yes	F2R
ENST00000319942	-1.72654	0.000542	0.045241	yes	RABGGTB
ENST00000320031	1.891648	2.13E-05	0.003717	yes	ITGA3
ENST00000320033	-1.58886	5.24E-06	0.001292	yes	ARL4D
ENST00000324873	2.170908	0.00033	0.0313	yes	NUPR1
ENST00000325617	1.134463	0.000579	0.047424	yes	CLGN
ENST00000325870	4.066089	6.03E-08	2.90E-05	yes	DMRTA1
ENST00000326335	2.663485	0.000266	0.026948	yes	CUL4A
ENST00000326840	2.438919	3.91E-11	4.85E-08	yes	DCBLD2
ENST00000327569	-1.60768	1.44E-05	0.00277	yes	ATP11C
ENST00000327741	-3.20853	1.24E-09	9.19E-07	yes	KRT81
ENST00000328300	1.193641	8.23E-06	0.00182	yes	COL4A5
ENST00000328736	2.019058	1.68E-05	0.003096	yes	PTPN21
ENST00000331442	-1.20821	0.000347	0.032343	yes	HIST1H1B
ENST00000331491	-1.3414	0.000216	0.022851	yes	HIST2H3D
ENST00000331666	12.10547	4.54E-07	0.00016	yes	EIF3C
ENST00000331817	-5.94001	2.52E-20	1.91E-16	yes	KRT7
ENST00000333151	-1.41137	3.77E-06	0.000953	yes	HIST1H2AJ
ENST00000333244	1.207257	0.000316	0.030399	yes	AHNAK2
ENST00000333493	-3.38518	4.51E-13	8.55E-10	yes	ADGRG3
ENST00000334504	1.023672	7.02E-05	0.009687	yes	COL4A6
ENST00000335183	-1.46766	0.00011	0.013636	yes	CDKN3
ENST00000335211	4.830357	8.72E-16	2.84E-12	yes	IGFN1
ENST00000336023	-1.18462	7.11E-05	0.009761	yes	TUBA1B
ENST00000336458	-1.25248	0.000288	0.028565	yes	EIF5A
ENST00000336596	-2.84831	4.86E-16	1.66E-12	yes	EPHA3
ENST00000338193	-2.54789	1.27E-05	0.002546	yes	PRIM1
ENST00000338380	-2.13879	1.65E-07	6.51E-05	yes	SLPI
ENST00000338530	1.388864	4.30E-06	0.001073	yes	MLPH
ENST00000338971	-11.5427	2.07E-06	0.000577	yes	SULT1A3
ENST00000340581	-9.84549	0.000267	0.026981	yes	ADGRG2
ENST00000340892	-1.8522	0.000468	0.040541	yes	TC2N
ENST00000340958	-8.94544	4.99E-10	4.26E-07	yes	CLDN4
ENST00000340965	-8.75933	3.08E-05	0.005063	yes	PAQR5

ENST00000341023	-1.52872	0.000133	0.015996	yes	HIST1H2AD
ENST00000341049	-1.46076	1.57E-05	0.002972	yes	CAV1
ENST00000341441	-10.8867	4.59E-08	2.31E-05	yes	MEST
ENST00000341524	-10.4818	4.96E-08	2.44E-05	yes	KCNAB2
ENST00000341627	1.466469	7.05E-05	0.009715	yes	TNFRSF12A
ENST00000342456	-1.16438	0.000257	0.02626	yes	ALDH3B1
ENST00000343304	-1.94885	8.04E-05	0.010711	yes	LRRC41
ENST00000343420	2.021452	5.00E-11	6.10E-08	yes	CD55
ENST00000345016	-11.079	3.32E-09	2.25E-06	yes	AP3D1
ENST00000345125	-10.9801	9.59E-08	4.07E-05	yes	CTSB
ENST00000347215	9.886713	2.30E-07	8.60E-05	yes	PMEPA1
ENST00000347343	-2.02574	1.80E-05	0.003253	yes	AURKA
ENST00000347800	10.31817	0.000143	0.017098	yes	ABCG1
ENST00000348581	-1.35394	0.000406	0.03622	yes	EXO1
ENST00000349945	1.639977	0.000327	0.031088	yes	NFAT5
ENST00000350997	-1.06411	0.000393	0.035475	yes	FADS1
ENST00000352433	-1.23026	0.000152	0.017935	yes	PTTG1
ENST00000354546	-1.50824	5.77E-06	0.001386	yes	ANXA6
ENST00000354624	3.577606	9.41E-10	7.30E-07	yes	HKDC1
ENST00000354675	1.752388	0.000402	0.035962	yes	AKAP12
ENST00000355057	-1.28611	0.000174	0.019664	yes	HIST1H4J
ENST00000355085	-1.79301	1.16E-06	0.00036	yes	C5AR1
ENST00000355086	1.714522	7.33E-08	3.34E-05	yes	SRGAP1
ENST00000355235	1.252989	0.000555	0.045918	yes	TBC1D9B
ENST00000355327	1.626347	9.51E-10	7.30E-07	yes	THSD4
ENST00000355991	8.748193	0.000159	0.018515	yes	FRMD8
ENST00000356530	-1.41064	1.60E-06	0.000468	yes	HIST1H2BF
ENST00000356698	1.801881	3.50E-08	1.81E-05	yes	RSPO3
ENST00000356936	-1.72751	5.91E-05	0.008419	yes	NCL
ENST00000357266	-1.84822	0.000401	0.035962	yes	FKBP5
ENST00000357370	2.211188	0.000184	0.020362	yes	NPIPB5
ENST00000357402	1.318947	1.59E-05	0.002987	yes	MVP
ENST00000357503	1.128185	0.000277	0.027617	yes	TOR4A
ENST00000357769	1.722824	2.32E-07	8.61E-05	yes	THSD4
ENST00000358739	-1.22532	0.000138	0.016586	yes	HIST1H2AI
ENST00000358913	2.702385	4.97E-05	0.007356	yes	MYPN
ENST00000359092	2.195889	3.05E-08	1.59E-05	yes	AXL
ENST00000359303	-1.69245	0.000135	0.01632	yes	HIST1H3J
ENST00000359543	-1.24289	0.000144	0.017106	yes	EMP2
ENST00000359546	-3.654	8.44E-27	1.44E-22	yes	CPLX2
ENST00000359579	-1.38834	3.31E-06	0.000853	yes	AKR1B10
ENST00000359611	-1.24968	6.78E-05	0.009436	yes	HIST1H2AM
ENST00000359949	-10.2527	2.47E-05	0.004163	yes	P2RX4
ENST00000360064	1.724366	1.17E-05	0.002412	yes	RYR2
ENST00000360190	-1.36159	7.71E-05	0.01037	yes	CDK5RAP2
ENST00000360301	-9.41645	0.000149	0.01758	yes	PPIP5K1
ENST00000360351	-1.35533	2.81E-05	0.004666	yes	MAP2
ENST00000360594	-1.96201	0.000115	0.014183	yes	TC2N
ENST00000360697	1.592745	1.70E-05	0.003107	yes	ADARB1
ENST00000361603	1.040353	7.71E-05	0.01037	yes	COL4A5
ENST00000361956	1.522041	1.34E-08	7.50E-06	yes	SMOC1
ENST00000361981	-1.8269	1.39E-06	0.000417	yes	L1CAM
ENST00000362096	1.439274	0.000122	0.014989	yes	UTY
ENST00000366548	-1.52699	1.08E-05	0.002275	yes	EXO1
ENST00000366637	7.84549	6.55E-08	3.09E-05	yes	DISC1
ENST00000366695	-1.69185	1.27E-07	5.18E-05	yes	HIST3H2A
ENST00000366741	-11.5395	5.90E-05	0.008419	yes	MRPL55
ENST00000367048	-1.33856	0.000122	0.01496	yes	ACAT2

ENST00000367063	2.030093	1.09E-10	1.18E-07	yes	CD55
ENST00000367064	1.790306	5.60E-10	4.66E-07	yes	CD55
ENST00000367067	1.845439	7.10E-06	0.001633	yes	CD55
ENST00000367142	-1.04389	0.000154	0.017986	yes	NUCKS1
ENST00000367157	-1.35155	0.000531	0.044757	yes	NUAK2
ENST00000367187	-1.23178	0.000326	0.031088	yes	PIK3C2B
ENST00000367466	-1.13566	0.000491	0.042099	yes	PLA2G4A
ENST00000367468	1.853085	5.55E-09	3.68E-06	yes	PTGS2
ENST00000367661	2.944727	2.19E-06	0.000605	yes	PAPPA2
ENST00000367718	10.00843	1.38E-07	5.57E-05	yes	TNFSF4
ENST00000368215	1.800657	3.48E-06	0.00089	yes	PTPRK
ENST00000368716	-1.08685	0.000515	0.043668	yes	S100A4
ENST00000368733	-12.2208	9.96E-06	0.002132	yes	S100A8
ENST00000368738	-5.73563	3.00E-15	8.53E-12	yes	S100A9
ENST00000368930	-10.0362	2.80E-05	0.004666	yes	CDC40
ENST00000368932	10.68825	3.48E-06	0.00089	yes	CDC40
ENST00000369158	-1.38489	7.75E-06	0.001731	yes	HIST2H3C
ENST00000369159	-1.10548	0.000168	0.019261	yes	HIST2H2AA3
ENST00000369163	-1.44833	1.65E-05	0.003073	yes	HIST1H3H
ENST00000369167	-1.15181	4.39E-05	0.006643	yes	HIST2H2BF
ENST00000369646	2.894818	5.17E-05	0.007573	yes	NT5E
ENST00000369651	4.769553	1.26E-06	0.000388	yes	NT5E
ENST00000369750	3.938599	4.54E-05	0.00682	yes	TPBG
ENST00000369880	-10.4164	5.10E-07	0.000176	yes	AS3MT
ENST00000370035	-1.30369	0.000478	0.041204	yes	FAM102B
ENST00000370103	-6.00375	1.36E-05	0.002673	yes	OLFM3
ENST00000370163	-8.53138	0.000613	0.049463	yes	AGL
ENST00000370315	-2.2062	0.000487	0.041812	yes	CGAS
ENST00000370501	3.083464	4.50E-14	1.06E-10	yes	NTSR1
ENST00000370765	1.225238	2.94E-05	0.004865	yes	DST
ENST00000371045	1.24341	5.62E-05	0.00807	yes	PDE4B
ENST00000371250	1.389392	6.39E-05	0.009005	yes	PLPP3
ENST00000371332	-10.1548	2.15E-06	0.000598	yes	HELLS
ENST00000371811	-10.1964	2.25E-06	0.000619	yes	IFIT3
ENST00000372216	1.30452	4.40E-05	0.006643	yes	COL4A6
ENST00000372764	1.877753	6.21E-10	5.11E-07	yes	PLAU
ENST00000372925	1.209955	0.000504	0.042944	yes	MACF1
ENST00000372991	-1.33001	0.000469	0.040594	yes	CCND3
ENST00000373176	-10.6227	2.26E-06	0.000619	yes	AK1
ENST00000373232	-1.07775	0.000298	0.029367	yes	PPA1
ENST00000373298	-4.65464	0.000535	0.044832	yes	ITM2A
ENST00000373701	1.153036	0.000132	0.01594	yes	OGT
ENST00000373970	4.108416	5.18E-10	4.37E-07	yes	DKK1
ENST00000374431	1.657488	0.000569	0.046843	yes	LPAR1
ENST00000374517	-1.06961	0.000256	0.026237	yes	TXN
ENST00000374694	1.386359	0.000215	0.022765	yes	FZD8
ENST00000374761	-9.12067	0.0001	0.012752	yes	RAP1GAP
ENST00000374811	-1.89819	9.31E-05	0.011996	yes	LAS1L
ENST00000374816	3.495411	0.000419	0.037117	yes	NRP1
ENST00000374822	2.866187	1.30E-14	3.42E-11	yes	NRP1
ENST00000374867	3.126623	4.09E-13	7.98E-10	yes	NRP1
ENST00000374875	3.586075	2.09E-17	9.02E-14	yes	NRP1
ENST00000374994	2.207436	1.01E-09	7.63E-07	yes	TGFBR1
ENST00000375064	8.866249	0.000172	0.019501	yes	TBC1D2
ENST00000375360	-10.9754	8.20E-14	1.81E-10	yes	PTPDC1
ENST00000376162	1.837774	7.64E-05	0.01031	yes	ITGBL1
ENST00000376447	3.946419	0.000153	0.017986	yes	RASEF
ENST00000376759	-1.14444	0.000588	0.048058	yes	RBM3

ENST00000377017	2.147946	4.89E-15	1.34E-11	yes	TIMP1
ENST00000377401	-1.36295	1.71E-05	0.003124	yes	HIST1H2BL
ENST00000377459	-1.58665	1.62E-06	0.00047	yes	HIST1H2AH
ENST00000377727	-1.44365	0.000138	0.016511	yes	HIST1H4H
ENST00000377791	-1.24023	1.45E-05	0.002784	yes	HIST1H2AC
ENST00000377819	9.936638	1.59E-08	8.74E-06	yes	RTN3
ENST00000377861	1.771961	8.92E-08	3.86E-05	yes	PCDH9
ENST00000378970	-1.15909	0.000222	0.023369	yes	NR0B1
ENST00000379361	2.479993	0.000269	0.02707	yes	PTCHD1
ENST00000379632	-10.3369	1.15E-05	0.002387	yes	BABAM2
ENST00000380060	2.087812	7.18E-08	3.31E-05	yes	NHS
ENST00000380554	-1.58662	3.14E-06	0.000819	yes	AKR1C3
ENST00000380779	3.497399	0.000235	0.024347	yes	MID1
ENST00000380790	10.40939	0.000118	0.014519	yes	YAF2
ENST00000380800	-2.28514	4.41E-05	0.006657	yes	BRWD1
ENST00000380956	-8.12928	0.000221	0.023319	yes	IRF4
ENST00000381055	2.432227	7.88E-10	6.34E-07	yes	ADAMTS6
ENST00000381280	1.43248	1.56E-07	6.18E-05	yes	SMOC1
ENST00000381312	-2.56895	9.68E-08	4.08E-05	yes	ADARB2
ENST00000381405	2.795895	7.89E-05	0.010547	yes	ESM1
ENST00000381577	2.60467	9.06E-12	1.29E-08	yes	CD274
ENST00000382199	2.194624	5.06E-05	0.007449	yes	IGF2BP2
ENST00000382297	1.782234	1.98E-05	0.00351	yes	KANK1
ENST00000382395	2.903716	2.47E-06	0.000662	yes	CPEB2
ENST00000382873	-1.84987	3.32E-05	0.005378	yes	FECH
ENST00000388738	3.244622	7.95E-05	0.010606	yes	KIAA1109
ENST00000388825	-1.7542	5.66E-07	0.000193	yes	GPX3
ENST00000389131	1.27865	5.32E-05	0.007743	yes	LOXL2
ENST00000389204	1.203189	0.000497	0.042458	yes	ASPH
ENST00000389658	1.477261	8.13E-08	3.61E-05	yes	LAMA1
ENST00000391921	2.101228	1.44E-11	1.93E-08	yes	CD55
ENST00000392067	9.351675	1.98E-07	7.60E-05	yes	SAMD4A
ENST00000393306	-1.42213	2.38E-06	0.000641	yes	OSGIN1
ENST00000393561	1.955434	1.21E-08	6.95E-06	yes	LAMB1
ENST00000393568	1.119347	2.12E-05	0.003712	yes	GALC
ENST00000393745	-3.65371	2.18E-11	2.81E-08	yes	CPLX2
ENST00000393760	-9.91189	0.000114	0.01412	yes	ZDHHC16
ENST00000393925	1.824277	3.91E-05	0.006102	yes	PHLDB2
ENST00000393955	-9.70275	0.000246	0.025322	yes	PC
ENST00000394066	9.276124	0.000182	0.020198	yes	KLC2
ENST00000394894	-1.19129	0.000232	0.024094	yes	KIF20A
ENST00000395135	-9.51175	0.000622	0.049969	yes	PML
ENST00000395254	2.412195	0.000233	0.024188	yes	ZNF365
ENST00000395288	-10.0038	5.82E-06	0.001386	yes	KBTBD4
ENST00000395606	1.732188	9.41E-05	0.012031	yes	DDHD1
ENST00000395711	10.37359	0.00028	0.027856	yes	SDAD1
ENST00000395956	-9.13785	0.000398	0.035749	yes	YWHAZ
ENST00000396087	-7.54432	0.000187	0.020542	yes	NAV2
ENST00000396595	11.59805	6.39E-05	0.009005	yes	LIMCH1
ENST00000397406	1.207393	0.000563	0.046447	yes	TSPAN4
ENST00000397560	1.576139	7.70E-06	0.00173	yes	KDM7A
ENST00000397707	8.594947	0.000131	0.01592	yes	SLC12A6
ENST00000397747	3.155627	2.21E-07	8.29E-05	yes	PDCD1LG2
ENST00000397843	1.375923	0.00021	0.022378	yes	ARHGEF12
ENST00000397909	1.867129	4.55E-07	0.00016	yes	NAV3
ENST00000399017	1.330404	6.15E-05	0.008718	yes	TTC3
ENST00000399494	-1.33137	5.89E-05	0.008419	yes	HMGB1
ENST00000400331	1.331975	3.79E-05	0.005976	yes	MACC1

ENST00000402174	8.92679	0.000335	0.031631	yes	ZNRF3
ENST00000403299	-8.39518	0.000317	0.030399	yes	CARD10
ENST00000403683	-1.38489	7.75E-06	0.001731	yes	HIST2H3A
ENST00000403729	1.977666	5.22E-07	0.000179	yes	ANTXR2
ENST00000404191	2.071628	2.30E-06	0.000628	yes	ANTXR2
ENST00000404648	-5.13516	3.22E-05	0.00526	yes	FGG
ENST00000404751	9.16323	0.000358	0.033059	yes	TRMT2A
ENST00000405460	8.094518	4.12E-05	0.006348	yes	ADGRV1
ENST00000406200	10.46489	4.31E-10	3.77E-07	yes	BCOR
ENST00000409057	-10.0038	0.00013	0.015749	yes	PBRM1
ENST00000409194	-1.69587	7.13E-06	0.001634	yes	ATP5MC3
ENST00000409315	-3.91702	4.36E-05	0.006641	yes	AGFG1
ENST00000409584	4.22668	5.20E-08	2.52E-05	yes	IL1RL1
ENST00000412568	8.321928	0.000458	0.039961	yes	PTPRF
ENST00000412585	1.44661	1.95E-06	0.000548	yes	HLA-B
ENST00000413802	2.944126	2.81E-05	0.004666	yes	NRP1
ENST00000414773	10.20864	0.000249	0.025535	yes	CLGN
ENST00000415314	-10.5507	0.000357	0.033018	yes	GPATCH4
ENST00000415759	-10.0679	2.66E-06	0.00071	yes	CEP78
ENST00000416334	4.275443	1.01E-05	0.002164	yes	NT5E
ENST00000418259	9.485158	5.98E-05	0.008485	yes	ATG2A
ENST00000418359	11.00609	2.21E-05	0.003827	yes	CNOT2
ENST00000418777	7.189825	1.71E-06	0.000487	yes	BNC2
ENST00000419255	2.36263	1.22E-08	6.97E-06	yes	SEMA3C
ENST00000419304	-1.43207	7.48E-06	0.001691	yes	AGR2
ENST00000421017	-1.07722	0.000351	0.032624	yes	ANXA2
ENST00000423313	-8.47843	0.00017	0.01936	yes	KIAA0040
ENST00000424270	11.45635	0.000183	0.020329	yes	SDCBP
ENST00000424424	2.169475	2.62E-09	1.79E-06	yes	PRNP
ENST00000425231	2.540902	5.92E-05	0.008419	yes	CDCP1
ENST00000425647	-1.9715	7.54E-07	0.000249	yes	CDK5RAP2
ENST00000426542	1.464978	0.000417	0.037068	yes	ARHGEF28
ENST00000428082	-11.359	8.68E-09	5.44E-06	yes	TVP23C
ENST00000428084	1.110101	0.000302	0.029584	yes	DOCK4
ENST00000428848	1.418268	2.02E-06	0.000565	yes	CDH13
ENST00000429972	2.155903	1.23E-13	2.63E-10	yes	FLNB
ENST00000431914	9.764872	0.000617	0.049735	yes	CTNNB1
ENST00000432372	3.333901	2.50E-05	0.00419	yes	NRP1
ENST00000432752	-9.77039	0.000232	0.024094	yes	DUS2
ENST00000433805	-11.0701	0.000371	0.034054	yes	COQ10A
ENST00000434452	-8.86625	0.000262	0.026619	yes	DSC3
ENST00000435572	-3.5927	1.31E-05	0.002594	yes	NFE2
ENST00000436190	-2.8499	0.000103	0.013065	yes	MAPRE2
ENST00000436759	10.73978	9.05E-26	1.24E-21	yes	PLEC
ENST00000436949	1.270036	2.47E-06	0.000662	yes	SEMA3A
ENST00000437131	10.78953	7.48E-07	0.000248	yes	GOLGA4
ENST00000438103	9.485158	0.000271	0.027215	yes	SBNO2
ENST00000438120	1.340489	1.51E-05	0.002885	yes	PTPRF
ENST00000438166	-1.64636	4.86E-06	0.001204	yes	GPD2
ENST00000438958	1.443683	0.000534	0.044832	yes	GOLGA8B
ENST00000439082	-1.15747	0.000125	0.015221	yes	AKR1C3
ENST00000439375	9.708509	0.000311	0.030203	yes	DNAH14
ENST00000440156	10.35902	3.18E-05	0.005219	yes	SLC4A7
ENST00000441883	12.16951	0.000154	0.018026	yes	MRPL28
ENST00000443272	-10.5539	2.02E-05	0.003539	yes	NFIC
ENST00000446158	11.65881	1.24E-05	0.0025	yes	EBP
ENST00000447185	-1.21674	0.000185	0.020469	yes	MAP2
ENST00000447266	-9.13785	0.000169	0.019356	yes	SENP6

ENST00000447280	1.12922	0.000205	0.021942	yes	SERPINE2
ENST00000448775	1.421323	1.68E-05	0.003096	yes	CLMP
ENST00000449103	1.134871	0.000307	0.029893	yes	PLXNB2
ENST00000449682	9.753774	1.06E-05	0.002258	yes	MST1
ENST00000449880	8.994353	3.86E-06	0.000972	yes	ITGB4
ENST00000450171	1.423548	0.000611	0.049459	yes	GRAMD1B
ENST00000450331	10.47506	1.17E-05	0.002412	yes	PNPLA6
ENST00000450366	-1.27015	8.92E-06	0.001946	yes	LANCL1
ENST00000450894	2.230062	6.15E-09	4.04E-06	yes	ITGB4
ENST00000453394	-6.14975	0.000595	0.048537	yes	RAD50
ENST00000456849	1.126154	0.000269	0.02707	yes	PAPSS2
ENST00000457286	-11.1943	0.000168	0.019261	yes	SNX8
ENST00000462132	-11.1434	1.40E-07	5.63E-05	yes	MEST
ENST00000465261	2.894597	1.50E-06	0.000442	yes	LMO7
ENST00000468300	-9.04985	7.52E-05	0.010215	yes	BRCA1
ENST00000472441	1.660471	5.29E-05	0.007719	yes	TM4SF1
ENST00000473453	2.276623	1.94E-05	0.003457	yes	TXNDC5
ENST00000473598	11.32755	1.92E-09	1.35E-06	yes	LMNA
ENST00000473702	10.95371	0.000185	0.020469	yes	TIPARP
ENST00000474594	-2.93797	3.96E-07	0.000143	yes	AHCYL2
ENST00000475377	9.60733	1.39E-06	0.000417	yes	FMNL2
ENST00000477813	-7.66178	2.48E-05	0.004163	yes	NAA50
ENST00000480683	6.174926	4.60E-05	0.006872	yes	KCNMA1
ENST00000486442	2.186512	3.08E-06	0.000806	yes	KLHL29
ENST00000491274	12.16428	0.000393	0.035475	yes	SDHB
ENST00000493452	6.248118	0.000197	0.021328	yes	FLNB
ENST00000494014	-2.97594	4.24E-12	6.43E-09	yes	EPHA3
ENST00000495646	-9.32193	0.000178	0.02008	yes	ASAP3
ENST00000497329	11.35535	0.000192	0.020964	yes	ANKRD36B
ENST00000504087	1.335676	1.19E-05	0.002441	yes	ANKRD50
ENST00000504228	9.37359	0.000257	0.02626	yes	KIAA1211
ENST00000504452	2.422984	5.41E-05	0.007832	yes	PRDM8
ENST00000507359	9.085694	3.26E-12	5.19E-09	yes	KCTD16
ENST00000508342	1.297023	1.27E-05	0.002546	yes	NEDD4
ENST00000511377	-4.05778	8.62E-09	5.44E-06	yes	CTNND2
ENST00000512044	2.488286	1.42E-06	0.000423	yes	DLC1
ENST00000513042	2.046997	6.45E-05	0.009042	yes	ARHGEF28
ENST00000515013	3.455004	1.80E-05	0.003253	yes	PRDM8
ENST00000515069	10.38082	6.36E-09	4.14E-06	yes	PLK4
ENST00000515641	1.453934	4.04E-05	0.006254	yes	ANKRD50
ENST00000517856	1.093607	3.90E-05	0.006099	yes	ASPH
ENST00000517905	8.792248	0.000547	0.045535	yes	ADAM19
ENST00000518291	-1.28666	0.000554	0.045902	yes	PAK3
ENST00000518659	4.008174	9.69E-05	0.012376	yes	TENM2
ENST00000521309	-12.1768	1.55E-05	0.002956	yes	YWHAZ
ENST00000521849	-3.1343	3.25E-05	0.005298	yes	FADS2
ENST00000522056	-2.00495	7.57E-06	0.001705	yes	FADS2
ENST00000524463	1.097397	8.72E-05	0.011461	yes	CTSC
ENST00000524581	10.16323	0.000202	0.021711	yes	EPB41L2
ENST00000525344	2.324631	7.46E-09	4.76E-06	yes	EXPH5
ENST00000525849	-5.45943	0.000531	0.044757	yes	EYA4
ENST00000526005	9.691162	7.17E-05	0.009814	yes	KBTBD4
ENST00000526202	2.251972	1.14E-06	0.000354	yes	LMO7
ENST00000526601	10.83763	4.39E-06	0.001091	yes	ZNF195
ENST00000526634	-11.0038	6.57E-07	0.000221	yes	LGALS8
ENST00000527096	1.14009	0.000537	0.044872	yes	PLEC
ENST00000530275	1.971525	2.43E-08	1.28E-05	yes	MACF1
ENST00000532462	10.08569	0.000402	0.035962	yes	PYGL

ENST00000534831	2.118979	1.27E-06	0.000391	yes	GPRC5A
ENST00000535015	1.362514	0.000169	0.01928	yes	ADGRD1
ENST00000536525	3.718818	3.12E-10	2.84E-07	yes	NAV3
ENST00000539372	12.88162	2.29E-09	1.58E-06	yes	TNFRSF1A
ENST00000539945	12.46998	5.29E-06	0.001292	yes	MDH1
ENST00000540351	1.271461	0.000156	0.018227	yes	UBC
ENST00000540499	-1.71268	0.000122	0.014989	yes	IDH2
ENST00000540630	3.10343	3.30E-05	0.005356	yes	MYPN
ENST00000541199	-12.7056	1.85E-08	1.00E-05	yes	ADGRG6
ENST00000541630	2.072261	0.000111	0.01371	yes	RAN
ENST00000541714	-8.81378	0.000262	0.026619	yes	TJP3
ENST00000541790	-1.13249	0.000343	0.032182	yes	HIST1H2BG
ENST00000541916	1.34187	1.24E-05	0.0025	yes	FNDC3A
ENST00000542053	10.73132	2.34E-08	1.25E-05	yes	NOL8
ENST00000542783	-9.02237	0.000109	0.013603	yes	TIPARP
ENST00000543496	3.738035	4.00E-13	7.98E-10	yes	TPBG
ENST00000544039	-4.74846	0.000288	0.028582	yes	ABCC9
ENST00000544246	1.575502	1.43E-05	0.00277	yes	PCDH9
ENST00000544915	4.244472	1.20E-05	0.002459	yes	ARNTL2
ENST00000545560	1.156563	0.000141	0.016923	yes	ITGBL1
ENST00000545683	-1.678	1.82E-05	0.003278	yes	HIST2H2BF
ENST00000545689	11.73697	8.20E-08	3.61E-05	yes	COL4A6
ENST00000545693	-8.86625	0.000455	0.03982	yes	PARD3
ENST00000546206	11.78136	0.000395	0.035511	yes	ARL6IP1
ENST00000546939	1.045247	0.000346	0.032334	yes	CD63
ENST00000549442	-3.11635	0.000576	0.047314	yes	HVCN1
ENST00000550253	1.914429	2.67E-08	1.40E-05	yes	TGFBR1
ENST00000550767	-1.51812	7.49E-05	0.010204	yes	TUBA1A
ENST00000551524	-11.7566	0.000123	0.014997	yes	NAP1L1
ENST00000552192	1.535805	2.91E-06	0.000767	yes	SLC6A15
ENST00000552516	1.518062	6.19E-07	0.00021	yes	TGFBR1
ENST00000556224	-9.77588	2.00E-05	0.003525	yes	HECTD1
ENST00000558745	-1.94794	0.000111	0.013731	yes	TSPAN3
ENST00000559209	9.268347	0.000244	0.025108	yes	RNF111
ENST00000559313	1.331871	0.000228	0.023776	yes	CCDC9B
ENST00000559420	1.367448	4.08E-05	0.0063	yes	GLCE
ENST00000563965	-2.70463	2.00E-05	0.003523	yes	STRA6
ENST00000566501	1.688418	6.05E-06	0.001425	yes	EIF3C
ENST00000567109	1.118229	0.000146	0.017343	yes	CDH13
ENST00000568219	-9.71996	0.000259	0.026368	yes	PALB2
ENST00000570272	-6.61738	1.38E-09	9.91E-07	yes	CCPG1
ENST00000570836	-8.94642	0.000323	0.030922	yes	ALOX15
ENST00000572789	11.32942	0.000589	0.048058	yes	PHF23
ENST00000578186	-1.35889	1.69E-05	0.003101	yes	HIST2H4A
ENST00000579662	3.133937	5.16E-19	3.44E-15	yes	ITGB4
ENST00000580070	11.48684	4.80E-05	0.00713	yes	NCOA4
ENST00000580974	2.571023	9.89E-09	6.05E-06	yes	PITPNC1
ENST00000584306	9.70275	0.000174	0.019664	yes	LRRC37A3
ENST00000588750	-1.9867	0.000393	0.035475	yes	APOC1
ENST00000590151	-3.84395	0.000611	0.049459	yes	ACLY
ENST00000590474	-1.32331	0.000188	0.020691	yes	MAP2K6
ENST00000592947	1.366013	3.71E-05	0.005884	yes	FSTL3
ENST00000597224	-1.81659	0.000179	0.02008	yes	FBL
ENST00000600873	-1.05526	0.000316	0.030399	yes	HNRNPL
ENST00000603017	-2.23597	6.54E-08	3.09E-05	yes	RASL10B
ENST00000604524	-9.39518	1.27E-07	5.18E-05	yes	ZSWIM8
ENST00000607355	-1.34643	5.29E-06	0.001292	yes	HIST2H2AA4
ENST00000610579	1.116866	0.000386	0.035144	yes	FURIN

ENST00000610854	-1.46301	5.29E-06	0.001292	yes	NEFL
ENST00000611185	12.51833	8.81E-08	3.83E-05	yes	RNF145
ENST00000612898	-1.19011	0.000175	0.019805	yes	HIST1H2BN
ENST00000613174	-1.60864	9.06E-06	0.001965	yes	HIST1H2AL
ENST00000613873	-10.2368	7.73E-05	0.01037	yes	MAP2K6
ENST00000614960	9.871392	1.45E-05	0.002784	yes	KIF14
ENST00000615871	8.759333	5.37E-05	0.007794	yes	AUTS2
ENST00000616167	-9.81911	3.55E-06	0.000905	yes	NCOA6
ENST00000616182	-1.25773	0.000354	0.032851	yes	HIST1H2BO
ENST00000616939	-11.182	6.10E-08	2.91E-05	yes	GPN1
ENST00000617492	11.30112	2.63E-07	9.60E-05	yes	INTS2
ENST00000618048	10.14636	0.000203	0.021766	yes	KCNMA1
ENST00000618052	-1.32248	1.32E-05	0.002607	yes	HIST1H3F
ENST00000618822	9.649855	0.000181	0.020159	yes	MAP7
ENST00000619128	11.11418	1.37E-05	0.002688	yes	PSMD1
ENST00000620417	-5.73335	0.000311	0.030203	yes	ARHGAP23
ENST00000620436	6.754888	1.08E-08	6.43E-06	yes	SLC16A6
ENST00000620755	1.413044	0.000341	0.032044	yes	NOMO1
ENST00000621112	-1.85488	1.72E-06	0.000487	yes	HIST1H2BM
ENST00000621150	-10.0589	0.000369	0.033913	yes	TNFAIP3
ENST00000621876	-11.1378	2.98E-06	0.000782	yes	KBTBD2
ENST00000621976	4.741467	4.99E-05	0.007362	yes	KCNQ3
ENST00000622089	8.878051	3.82E-18	2.01E-14	yes	ITSN2
ENST00000622132	10.47168	2.19E-10	2.21E-07	yes	ABCB1
ENST00000622220	-11.3862	1.91E-05	0.003417	yes	RNFT2
ENST00000622629	-9.38802	1.14E-12	1.99E-09	yes	MSH6
ENST00000623361	9.770389	2.20E-09	1.53E-06	yes	IQCE
ENST00000626301	-10.3916	9.45E-07	0.000299	yes	DDX3X
ENST00000626972	-9.41645	0.000616	0.049712	yes	CCT8
ENST00000627032	-10.0589	5.47E-05	0.007886	yes	GLG1
ENST00000628426	-10.9967	7.12E-12	1.06E-08	yes	CHST15
ENST00000631006	1.171443	8.97E-05	0.011667	yes	SRGAP1
ENST00000633646	1.742975	1.40E-05	0.002723	yes	GRAMD1B
ENST00000635200	-1.4131	8.95E-07	0.000286	yes	AL031777.3
ENST00000635491	-1.30912	0.000326	0.031088	yes	HIST1H4H
ENST00000635736	1.179169	2.34E-05	0.004009	yes	GRAMD1B
ENST00000636400	8.451211	2.31E-06	0.00063	yes	TCF4
ENST00000636933	1.437649	0.000305	0.029708	yes	PLCXD2
ENST00000637017	-7.20457	1.03E-12	1.85E-09	yes	ZNF831
ENST00000637161	10.17576	8.55E-05	0.011277	yes	EHMT1
ENST00000638295	-9.23681	0.000508	0.043128	yes	SCARB2
ENST00000639244	-6.45943	0.000195	0.021206	yes	SCN3A
ENST00000639525	9.430453	0.000542	0.045241	yes	SPIN3
ENST00000639850	-9.54432	0.000425	0.03755	yes	ME2
ENST00000639952	3.825426	5.79E-06	0.001386	yes	KCNH1
ENST00000640614	10.03617	0.000543	0.045271	yes	RBCK1
ENST00000642627	8.24476	7.00E-06	0.001616	yes	TSC1
ENST00000642864	7.886713	0.000215	0.02279	yes	IQSEC2
ENST00000644074	9.613483	0.000162	0.018703	yes	DDX3X
ENST00000644155	-8.30682	6.64E-05	0.009279	yes	PPFIA1
ENST00000644836	2.075334	9.00E-10	7.06E-07	yes	CD55
ENST00000644934	-1.38993	1.96E-05	0.003471	yes	GPI
ENST00000645114	1.185097	4.39E-05	0.006643	yes	DYNC1H1
ENST00000645211	1.128419	0.000215	0.022765	yes	ADGRV1
ENST00000645234	-10.303	1.80E-06	0.000508	yes	CSNK2A1
ENST00000645323	2.547377	4.84E-10	4.19E-07	yes	CD55
ENST00000645356	-11.3068	1.30E-06	0.000393	yes	SOX9
ENST00000645564	9.941537	1.96E-05	0.003471	yes	DEPDC5

ENST00000646146	2.486962	3.43E-10	3.08E-07	yes	GRAMD1B
ENST00000646548	4.595532	1.29E-06	0.000392	yes	DYRK1A
TCONS_00002063	1.228066	0.000578	0.047419	yes	PTPRF
TCONS_00002409	8.946419	0.000379	0.034573	yes	RNF11
TCONS_00003483	10.05889	3.04E-10	2.81E-07	yes	TMEM56
TCONS_00003711	8.906891	1.27E-05	0.002546	yes	AMY2B
TCONS_00006135	2.052721	1.68E-10	1.80E-07	yes	-
TCONS_00006140	1.960583	1.10E-08	6.43E-06	yes	-
TCONS_00006147	2.055894	4.39E-08	2.24E-05	yes	C14orf178
TCONS_00006165	2.990419	2.11E-17	9.02E-14	yes	PAPPA2
TCONS_00006221	9.702173	1.16E-09	8.73E-07	yes	TOR3A
TCONS_00006500	-9.60733	1.61E-05	0.003008	yes	PLA2G4A
TCONS_00006768	9.058894	0.000369	0.033913	yes	-
TCONS_00006906	2.871014	6.48E-05	0.009071	yes	Igfn1
TCONS_00006910	2.62532	2.35E-05	0.004009	yes	IGFN1
TCONS_00007236	2.349528	3.84E-05	0.006051	yes	CD55
TCONS_00007858	-1.8977	2.00E-07	7.64E-05	yes	Hist3h2bb
TCONS_00008098	1.533247	2.56E-07	9.42E-05	yes	RYR2
TCONS_00008158	-1.51335	1.09E-05	0.002282	yes	EXO1
TCONS_00008834	7.803055	0.000312	0.030203	yes	PLEKHG5
TCONS_00009331	4.345775	0.000146	0.017336	yes	SH2D5
TCONS_00010733	1.25856	0.000174	0.019683	yes	LRP8
TCONS_00011306	8.855907	0.000579	0.047424	yes	SSX2IP
TCONS_00011317	9.416446	3.54E-05	0.00563	yes	MCOLN3
TCONS_00011556	-10.8271	3.00E-10	2.81E-07	yes	ABCA4
TCONS_00012893	1.466537	1.62E-06	0.00047	yes	-
TCONS_00013638	-11.1378	8.13E-08	3.61E-05	yes	ADAR
TCONS_00014460	2.534922	1.09E-08	6.43E-06	yes	-
TCONS_00014461	1.384763	7.34E-06	0.001671	yes	-
TCONS_00014462	3.311586	1.45E-15	4.30E-12	yes	-
TCONS_00014463	1.618758	4.77E-07	0.000167	yes	-
TCONS_00014464	1.136705	0.000254	0.026015	yes	-
TCONS_00014791	9.437405	0.000196	0.021261	yes	UCHL5
TCONS_00016361	-1.21996	9.82E-05	0.01252	yes	AKR1C3
TCONS_00016999	3.788496	1.67E-05	0.003096	yes	-
TCONS_00018549	9.550747	0.000189	0.020759	yes	Zdhhc16
TCONS_00018695	1.745322	0.000226	0.023626	yes	SFXN3
TCONS_00019224	1.469485	0.000168	0.019261	yes	-
TCONS_00020174	1.624491	0.000168	0.019261	yes	-
TCONS_00020321	1.146572	7.55E-05	0.010239	yes	ITGB1
TCONS_00020342	4.052321	1.04E-06	0.000326	yes	NRP1
TCONS_00020750	1.484034	1.20E-05	0.002459	yes	PGBD3
TCONS_00022220	11.37902	1.11E-07	4.58E-05	yes	LDB1
TCONS_00022532	8.84549	3.40E-12	5.28E-09	yes	CACUL1
TCONS_00023420	9.871392	0.00055	0.04562	yes	TUB
TCONS_00025003	7.92679	0.000312	0.030203	yes	RPLP0
TCONS_00025912	-8.24476	0.000267	0.026957	yes	ANO1
TCONS_00025928	9.129283	1.71E-06	0.000487	yes	PPFIA1
TCONS_00026935	2.688374	2.68E-10	2.54E-07	yes	DDX10
TCONS_00027481	5.664612	0.000416	0.03702	yes	GRAMD1B
TCONS_00027608	-10.6408	0.000487	0.041812	yes	HYLS1
TCONS_00028887	-2.82608	2.57E-11	3.25E-08	yes	USH1C
TCONS_00030010	-1.1704	8.83E-05	0.011505	yes	FADS1
TCONS_00031361	1.928809	0.000535	0.044832	yes	-
TCONS_00032538	9.72565	0.000501	0.042791	yes	SIK3
TCONS_00033084	8.67948	9.34E-05	0.011996	yes	ARHGAP32
TCONS_00035152	3.181702	9.12E-05	0.011798	yes	DAZAP2
TCONS_00035391	1.513704	6.91E-05	0.009548	yes	HNRNPA1

TCONS_00035968	-2.51725	0.00016	0.018628	yes	CAND1
TCONS_00035974	2.524955	0.000143	0.01707	yes	-
TCONS_00037715	6.736966	0.000565	0.046565	yes	TMEM132B
TCONS_00038229	9.146357	0.00035	0.032537	yes	NOP2
TCONS_00040192	-8.89684	3.43E-05	0.005498	yes	MAP3K12
TCONS_00041622	1.223754	2.21E-05	0.003827	yes	NTN4
TCONS_00041869	7.906891	1.72E-05	0.003131	yes	NUAK1
TCONS_00041870	-7.88671	2.45E-05	0.004144	yes	NUAK1
TCONS_00042186	-2.67957	4.24E-05	0.006517	yes	RASAL1
TCONS_00043357	1.922166	4.35E-05	0.006641	yes	DNAJC15
TCONS_00043957	2.4359	0.000387	0.035193	yes	LMO7
TCONS_00044911	9.179909	8.83E-05	0.011505	yes	SLC7A1
TCONS_00045000	1.755816	6.19E-06	0.001449	yes	N4BP2L2
TCONS_00045008	-10.28	0.000366	0.03374	yes	N4BP2L2
TCONS_00050252	9.366322	7.13E-11	8.40E-08	yes	HECTD1
TCONS_00050709	10.92926	0.000147	0.017367	yes	FERMT2
TCONS_00051321	9.808428	1.39E-05	0.00272	yes	ELMSAN1
TCONS_00051341	-9.00375	0.000105	0.013187	yes	ENTPD5
TCONS_00053706	-7.87036	1.60E-08	8.76E-06	yes	CGNL1
TCONS_00054946	7.118941	8.19E-07	0.000266	yes	GOLGA6L9
TCONS_00055904	2.161463	0.000202	0.021722	yes	GOLGA8A
TCONS_00056455	1.120159	0.000392	0.035475	yes	FBN1
TCONS_00058381	9.775885	1.87E-07	7.31E-05	yes	TTC23
TCONS_00059466	7.803055	4.04E-05	0.006254	yes	MKL2
TCONS_00060527	9.329423	8.16E-05	0.010824	yes	ZNF646
TCONS_00060549	1.180744	0.000463	0.04032	yes	FUS
TCONS_00064285	1.025653	0.000548	0.045535	yes	SMG1
TCONS_00064551	1.169165	0.000342	0.032107	yes	SIAH1
TCONS_00066552	-8.59495	6.91E-06	0.0016	yes	KIF1C
TCONS_00067602	-1.81168	5.14E-05	0.007556	yes	KSR1
TCONS_00068060	-11.6809	1.30E-05	0.002593	yes	TAF15
TCONS_00068847	-11.3952	7.11E-13	1.31E-09	yes	FMNL1
TCONS_00073973	10.13785	3.26E-05	0.005309	yes	MRPL45
TCONS_00074241	9.444325	0.000322	0.030854	yes	ANKRD40
TCONS_00074718	10.88671	0.00053	0.044757	yes	POLG2
TCONS_00076347	3.264703	0.000181	0.020153	yes	ANKRD20A4
TCONS_00076348	3.503403	5.53E-06	0.001335	yes	ANKRD20A8P
TCONS_00076354	3.962974	1.10E-08	6.43E-06	yes	ANKRD20A4
TCONS_00076376	6.438792	3.67E-06	0.000931	yes	CYP4F3
TCONS_00077750	1.472816	3.44E-05	0.005498	yes	-
TCONS_00077815	1.577336	2.27E-05	0.003915	yes	LAMA1
TCONS_00078099	2.533432	4.35E-05	0.006641	yes	ANKRD29
TCONS_00078172	-9.84549	2.56E-07	9.42E-05	yes	CDH2
TCONS_00079331	-8.94642	8.32E-08	3.64E-05	yes	ZNF516
TCONS_00080925	1.521985	9.89E-05	0.012584	yes	SIN3B
TCONS_00083043	2.637956	0.000382	0.034824	yes	FOSB
TCONS_00083375	1.5184	0.000125	0.015221	yes	PPP1R15A
TCONS_00084713	7.78136	0.00018	0.020153	yes	MIER2
TCONS_00088087	1.431643	5.53E-05	0.007951	yes	PLAUR
TCONS_00088164	-8.07682	4.83E-07	0.000168	yes	ZNF229
TCONS_00088165	8.491853	1.33E-08	7.50E-06	yes	ZNF229
TCONS_00088320	10.04075	9.92E-09	6.05E-06	yes	SYMPK
TCONS_00089677	-9.51175	0.000302	0.029584	yes	ZBTB45
TCONS_00090576	8.84549	0.000354	0.032851	yes	LTBP1
TCONS_00090986	3.534497	3.39E-05	0.005466	yes	PRKCE
TCONS_00091369	10.28	5.47E-05	0.007886	yes	LGALSL
TCONS_00091769	1.91908	1.25E-09	9.19E-07	yes	TIGD1
TCONS_00092878	1.318977	0.000201	0.021702	yes	DPP10

TCONS_00093253	9.351675	8.03E-11	9.14E-08	yes	Nbea
TCONS_00093423	1.340911	1.15E-05	0.002389	yes	SPOPL
TCONS_00093598	2.685364	0.000241	0.024901	yes	MBD5
TCONS_00093663	-8.835	4.18E-06	0.001049	yes	FMNL2
TCONS_00093767	-8.40282	0.000303	0.029646	yes	Psmd14
TCONS_00095110	6.539159	7.95E-07	0.00026	yes	NANOGNB
TCONS_00095223	11.20049	0.000269	0.02707	yes	CPS1
TCONS_00096408	8.212699	7.12E-05	0.009761	yes	SNED1
TCONS_00097729	6.736966	1.93E-05	0.003454	yes	SLC8A1
TCONS_00098157	12.06788	8.74E-05	0.011461	yes	PSME4
TCONS_00100485	2.89921	1.11E-18	6.34E-15	yes	-
TCONS_00101162	1.935725	8.94E-10	7.06E-07	yes	Pol
TCONS_00101235	11.33129	2.05E-07	7.76E-05	yes	ORC4
TCONS_00102399	1.456065	1.11E-05	0.002313	yes	NEMP2
TCONS_00102521	1.796793	4.45E-07	0.000158	yes	SF3B1
TCONS_00103962	-1.38611	0.000295	0.029066	yes	NDUFA10
TCONS_00104030	-5.29462	4.69E-05	0.006984	yes	MTERF4
TCONS_00104851	-2.42146	1.57E-05	0.002972	yes	GVQW1
TCONS_00105703	-1.45846	1.40E-05	0.002724	yes	CSE1L
TCONS_00106572	1.487741	0.000214	0.022755	yes	GPCPD1
TCONS_00107240	-10.7759	2.13E-10	2.21E-07	yes	RBM12
TCONS_00107375	-3.6937	0.000168	0.019261	yes	TGM2
TCONS_00107473	3.915111	2.13E-08	1.15E-05	yes	-
TCONS_00107930	10.42347	0.000238	0.024657	yes	AURKA
TCONS_00108427	2.798947	6.82E-08	3.19E-05	yes	GVQW1
TCONS_00108558	-2.5679	2.20E-10	2.21E-07	yes	NCAM2
TCONS_00109339	-9.69697	0.000106	0.01327	yes	PCBP3
TCONS_00110545	7.67948	3.22E-06	0.000838	yes	PTTG1IP
TCONS_00111748	1.361242	0.000131	0.015908	yes	HMOX1
TCONS_00113903	-1.89108	1.04E-05	0.002209	yes	ST13
TCONS_00113993	-11.0154	4.06E-21	3.47E-17	yes	TCF20
TCONS_00114325	8.691162	0.000265	0.02685	yes	CHL1
TCONS_00115987	1.682375	3.67E-05	0.005831	yes	DOCK3
TCONS_00117805	6.584963	2.97E-07	0.000108	yes	PHLDB2
TCONS_00117826	1.448184	0.000468	0.040541	yes	TTMP
TCONS_00117847	10.74259	1.40E-12	2.39E-09	yes	SLC35A5
TCONS_00118606	8.714246	9.09E-05	0.011779	yes	ARMC8
TCONS_00119032	1.223903	6.45E-05	0.009042	yes	AADAC
TCONS_00119033	1.891033	3.62E-10	3.22E-07	yes	AADAC
TCONS_00120779	-10.0544	0.000289	0.028592	yes	PIGX
TCONS_00121538	8.805744	2.37E-14	5.79E-11	yes	TOP2B
TCONS_00122007	3.139923	5.55E-23	5.41E-19	yes	CDCP1
TCONS_00122624	-5.79442	0.000444	0.038945	yes	PBRM1
TCONS_00123332	2.196347	1.00E-08	6.05E-06	yes	DCBLD2
TCONS_00123673	9.321928	1.48E-07	5.90E-05	yes	-
TCONS_00123921	-3.65243	4.38E-05	0.006643	yes	HGD
TCONS_00124994	2.447881	0.000145	0.017224	yes	CCNL1
TCONS_00126002	1.99084	4.94E-05	0.007327	yes	ATP13A3
TCONS_00126271	8.438792	2.38E-05	0.004058	yes	KIAA0226
TCONS_00126699	3.043345	1.42E-15	4.30E-12	yes	KIAA0232
TCONS_00127328	1.229276	0.000101	0.012831	yes	-
TCONS_00127360	9.031586	7.71E-08	3.49E-05	yes	-
TCONS_00127684	1.525584	8.80E-05	0.011505	yes	DCAF4L1
TCONS_00128193	-1.59712	8.72E-05	0.011461	yes	-
TCONS_00128230	4.721761	0.000375	0.034286	yes	MTHFD2L
TCONS_00129198	7.78136	0.000105	0.013192	yes	FGF2
TCONS_00129335	-6.57851	2.33E-10	2.28E-07	yes	JADE1
TCONS_00131451	1.766636	3.27E-06	0.000847	yes	C16orf89

TCONS_00132453	7.691162	6.57E-06	0.001527	yes	UBA6
TCONS_00132644	2.227177	1.33E-09	9.67E-07	yes	-
TCONS_00132646	3.011405	4.85E-08	2.40E-05	yes	ERVK13-1
TCONS_00132815	3.467868	1.03E-16	3.92E-13	yes	ANTXR2
TCONS_00132826	2.848538	4.92E-07	0.000171	yes	-
TCONS_00133947	4.770074	0.00019	0.020759	yes	C16orf89
TCONS_00133953	3.481955	3.76E-17	1.51E-13	yes	-
TCONS_00134083	2.468769	4.78E-08	2.38E-05	yes	TIGD1
TCONS_00135482	1.569251	1.04E-06	0.000326	yes	-
TCONS_00135514	6.523562	9.63E-09	5.98E-06	yes	TIGD1
TCONS_00135572	-2.82623	1.43E-06	0.000425	yes	NPR3
TCONS_00135705	10.52813	3.90E-05	0.006099	yes	SLC1A3
TCONS_00135778	8.770389	4.02E-05	0.006252	yes	PTGER4
TCONS_00137042	-10.8559	8.79E-12	1.28E-08	yes	CAST
TCONS_00137275	7.464886	4.13E-07	0.000149	yes	-
TCONS_00137777	8.437405	0.000438	0.038488	yes	IL13
TCONS_00139456	-3.62265	0.000304	0.029708	yes	Cplx2
TCONS_00139661	-7.71425	0.000106	0.013293	yes	ZNF354C
TCONS_00139717	-5.31832	9.66E-06	0.002079	yes	PKP2
TCONS_00140061	-5.2854	4.32E-08	2.22E-05	yes	CTNND2
TCONS_00141476	1.968919	0.00033	0.0313	yes	ADAMTS6
TCONS_00142774	-2.7623	7.24E-08	3.32E-05	yes	MCC
TCONS_00143758	1.921715	0.000179	0.02008	yes	-
TCONS_00143768	1.277825	5.33E-05	0.007743	yes	-
TCONS_00144009	-1.68708	9.33E-05	0.011996	yes	ANXA6
TCONS_00144280	8.146357	0.000521	0.044058	yes	PANK3
TCONS_00144350	3.373458	3.35E-05	0.005404	yes	GVQW1
TCONS_00145016	4.61471	0.000561	0.046372	yes	BMP6
TCONS_00145374	-1.1824	0.000166	0.019136	yes	Hist1h2ab
TCONS_00145383	-1.39202	9.68E-06	0.002079	yes	HIST1H2BH
TCONS_00145458	11.07236	6.87E-08	3.19E-05	yes	HMGN4
TCONS_00145513	-1.80778	6.27E-07	0.000212	yes	HIST1H2BO
TCONS_00146873	8.67948	0.000179	0.02008	yes	PHF3
TCONS_00146914	8.643856	1.33E-05	0.002629	yes	FAM135A
TCONS_00146998	-9.32942	2.00E-10	2.10E-07	yes	CD109
TCONS_00148070	1.811252	6.13E-06	0.00144	yes	RSPO3
TCONS_00148305	4.785725	0.000496	0.042458	yes	-
TCONS_00148327	-11.2448	1.67E-06	0.000482	yes	TNFAIP3
TCONS_00148378	3.052852	7.63E-10	6.21E-07	yes	-
TCONS_00149435	2.037755	0.000317	0.030399	yes	ZNF714
TCONS_00149979	-1.85094	0.000555	0.045918	yes	ZSCAN31
TCONS_00150252	11.91563	1.66E-05	0.003078	yes	DDX39B
TCONS_00151136	2.893085	4.56E-08	2.31E-05	yes	PTCHD4
TCONS_00151139	1.450661	7.39E-05	0.010101	yes	PTCHD4
TCONS_00151343	7.588715	7.57E-11	8.77E-08	yes	DST
TCONS_00151351	1.231165	0.000187	0.020542	yes	DST
TCONS_00152742	3.114016	0.000153	0.017944	yes	-
TCONS_00153353	1.476737	0.000172	0.019501	yes	HIVEP2
TCONS_00153560	1.277745	9.00E-06	0.001959	yes	SYNE1
TCONS_00156620	1.518497	4.25E-05	0.006525	yes	-
TCONS_00158909	2.03858	7.08E-07	0.000236	yes	TNRC18
TCONS_00159368	1.347647	2.63E-05	0.004389	yes	-
TCONS_00160203	2.086936	0.000107	0.013334	yes	7-Sep
TCONS_00161102	2.315596	9.45E-06	0.002043	yes	SEMA3A
TCONS_00161470	1.180494	0.000341	0.032044	yes	gag-pol
TCONS_00161772	9.103288	0.000222	0.023369	yes	RASA4
TCONS_00162029	6.83289	1.08E-05	0.002275	yes	LAMB4
TCONS_00162698	1.750193	1.83E-07	7.19E-05	yes	DGKI

TCONS_00163752	2.429138	1.83E-12	3.05E-09	yes	-
TCONS_00163753	1.564878	7.60E-07	0.00025	yes	-
TCONS_00163793	8.965784	0.000223	0.023411	yes	MICU3
TCONS_00164557	8.691162	7.45E-06	0.001691	yes	AGPAT6
TCONS_00166281	-3.72008	0.000106	0.01327	yes	FAM83A
TCONS_00166282	-4.29739	2.36E-06	0.00064	yes	FAM83A
TCONS_00167409	1.537337	0.000195	0.021206	yes	CSGALNACT1
TCONS_00167410	1.772347	0.000305	0.029708	yes	CSGALNACT1
TCONS_00169529	5.900867	4.59E-05	0.006872	yes	CSMD3
TCONS_00171181	4.115477	5.09E-18	2.49E-14	yes	ZNF714
TCONS_00171435	8.770389	7.90E-08	3.55E-05	yes	-
TCONS_00174105	-3.91995	0.000432	0.038006	yes	NTNG2
TCONS_00174765	2.089662	5.01E-08	2.45E-05	yes	BNC2
TCONS_00174884	-6.98489	0.00034	0.032044	yes	elavl2
TCONS_00177218	-6.64386	0.000474	0.040981	yes	ASTN2
TCONS_00177449	8.60733	0.000413	0.036811	yes	ZBTB6
TCONS_00178069	-10.4979	0.000195	0.021206	yes	SAPCD2
TCONS_00178536	2.799821	9.48E-08	4.05E-05	yes	NHS
TCONS_00179909	7.491853	0.000419	0.037117	yes	DRP2
TCONS_00181015	-5.24793	0.000452	0.039552	yes	MTMR1
TCONS_00181130	8.866249	0.00023	0.024003	yes	ZNF275
TCONS_00181817	3.251918	0.000357	0.033021	yes	Pol
TCONS_00182485	-10.7313	4.80E-30	1.64E-25	yes	HUWE1
TCONS_00183260	2.086024	1.21E-05	0.002459	yes	ZMAT1
TCONS_00184163	4.113563	5.55E-19	3.44E-15	yes	-
TCONS_00184805	1.687117	8.89E-06	0.001946	yes	Pol
TCONS_00184830	1.237896	8.12E-05	0.010787	yes	Txlng
TCONS_00184849	1.157005	0.000336	0.031659	yes	TXLNG
